# Blockchain-Backed Revocation and Yang–Baxter Consistency Screening for Zero-Trust IoT Admission Control

**DOI:** 10.3390/s26185960

**Published:** 2026-09-20

**Authors:** Yair E. Rivera-Julio, Esmeide A. Leal-Narváez, Javier Prieto Tejedor

**Affiliations:** 1 Department of Computer Systems Engineering, Corporación Universitaria Americana, Barranquilla 080002, Colombia; 2 Department of Productivity and Data, Faculty of Engineering, Universidad Autónoma del Caribe, Barranquilla 080020, Colombia; esmeide.leal@uac.edu.co; 3 Department of Computer Science and Automation, Faculty of Science, University of Salamanca, 37008 Salamanca, Spain; javierp@usal.es

**Keywords:** Blockchains, smart contracts, IoT authentication, zero-trust access control, Yang–Baxter equation, proactive credential revocation, Merkle anchoring, decentralized identifiers (DID), permissioned ledgers, edge intelligence, TMTO chains, Brauer configuration algebra, secure telemetry, verifiable audit logs

## Abstract

IoT deployments handle credential hygiene reactively: cloned, replayed, or stale credentials are typically discovered only after misuse, and revocation state is often propagated through centralized lists whose integrity cannot be independently verified. This article introduces the Yang–Baxter IoT Consistency Gateway (YB-IoT-CG), a Zero-Trust admission-control framework that pushes an algebraic layer of credential screening to the edge and anchors security evidence on a modeled permissioned-ledger architecture. YB-IoT-CG operates after conventional secret-based authentication and Yang–Baxter equality is used as an execution-consistency invariant rather than as proof of device identity or message authenticity. Each authenticated message is hashed into a digest and reduced to an algebraic transcript that is verified over two Yang–Baxter traversal paths. Beyond equality checking, chain-proximity metrics inspired by time–memory trade-off analysis, nonce and timestamp freshness heuristics, and contextual risk scoring identify credentials that should be proactively challenged or revoked before telemetry is admitted. The proactive risk component is deterministic and policy-based rather than a learned predictive model. Decisions are batched into Merkle trees whose roots are represented through a permissioned-ledger model, credential revocation is propagated through a modeled on-chain registry, and device identities are bound to W3C Decentralized Identifiers (DIDs) with verifiable credentials. This design provides tamper-evident audit support while keeping ledger interaction off the packet decision path. Validation is based on a controlled Python 3.13.7 packet-level simulation and a parameterized ledger model, not on a physical IoT or live Hyperledger Fabric deployment. Across 60 seeded simulation runs, the complete post-authentication screening pipeline obtained a median incremental decision time of 7.8 μs, 99.4% aggregate decision accuracy for the modeled attack classes, a 0.43% false rejection rate, and a 31.4 ms amortized ledger service-time equivalent per decision for a batch size of 64. The 99.4% value is a property of the composed freshness/context/registry/YB policy and is not a YB-only detection rate; the YB-specific positive guarantee is limited to the isolated fault class of Proposition 7. These results provide simulation-based evidence supporting a lightweight, explainable, auditable, and proactive approach to credential hygiene at the edge.

## 1. Introduction

A contemporary setup could consist of dozens or more inexpensive microcontrollers operating sensor networks, smart meters, video cameras, actuators, programmable devices, and edge gateways. The devices communicate telemetry and commands to local displays, cloud services, digital representations, and blockchain databases. Therefore, the overall system’s security falls on whether each device can authenticate its identity, link session tokens to a unique random number, and send authenticated data without leaving reusable secrets open [1].

**The reactive credential-hygiene problem.** Most of the methods used for authentication in IoT depend on some mechanisms such as pre-shared keys, TLS certificates, JSON Web Tokens (JWTs), OAuth-style bearer tokens, device certificates, or lightweight message authentication codes. While these mechanisms are indisputably relevant, they do not solve the lifecycle challenge of weak, copied, expired, or colliding credentials. In many cases, the detection of a compromised credential is carried out through the identification of suspicious telemetry, failed login attempts, identical messages, or human review [2]. Moreover, even when a revocation decision is finally taken, its propagation across distributed gateways is often based on mutable, centralized lists whose integrity cannot be independently verified.

**Research gap and design position.** The objective of this work is not to replace established cryptographic authentication with an algebraic construction. In particular, Yang–Baxter consistency is not claimed to be superior to elliptic-curve cryptography, MACs, signatures, or certificates, because these mechanisms solve a different problem: they establish cryptographic authenticity from a secret or private key. YB-IoT-CG starts *after* that lower security layer has successfully verified the credential and adds a small, deterministic pre-admission consistency and risk-governance stage. The research gap addressed here is therefore the design of a low-complexity, explainable, edge-resident layer that can combine authenticated credential state, freshness, context, proactive revocation policy, and verifiable distributed audit without introducing a learning model or a synchronous blockchain operation into the packet path.

**From mathematical consistency to post-authentication execution checking.** The Yang–Baxter equation (YBE) expresses a consistency condition in which two ordered traversals of the same triple yield the same final state. In YB-IoT-CG this equality is *not* an authentication criterion and is not claimed to detect an externally modified identifier, token, nonce, or payload when both paths are recomputed correctly. Cryptographic authenticity is established exclusively by the preceding verifier ${\mathrm{Auth}}_{0}$. The YB stage is retained as a redundant execution-consistency check: under the isolated single-path perturbation model formalized in Proposition 7, a nonzero corruption of one intermediate state produces a path mismatch. Replay, clone, stale-message, and contextual anomalies are handled by the freshness/context/registry policy rather than by Yang–Baxter divergence [3].

The word “authentication” in the preceding architectural description is layered: cryptographic authenticity is supplied by the preceding MAC/signature/certificate verifier, denoted ${\mathrm{Auth}}_{0}$, whereas Yang–Baxter equality is used only as an additional deterministic execution-consistency invariant. As proved in Section 5, every honestly recomputed external input remains Yang–Baxter consistent for the instantiated map; the positive mismatch guarantee is therefore limited to the declared isolated intermediate-state perturbation model.

**Proactive revocation.** In addition to the execution-consistency check, the proposed scheme applies a deterministic policy score that combines freshness, context, and an administratively defined endpoint-proximity heuristic. The TMTO-inspired component is used only as a compact indexing and policy mechanism; geometric proximity after hashing/reduction is not asserted to have cryptanalytic or probabilistic significance for credential compromise. If the aggregate score crosses a configured threshold, the gateway challenges or, when corroborating evidence is present, revokes the credential before telemetry admission. Thus, “proactive” denotes pre-admission policy enforcement rather than prediction of compromise [4].

The term *proactive* is used in a deterministic policy sense. The risk stage does not train a predictor, infer learned labels, or estimate a machine-learning probability of compromise; it applies predeclared endpoint, freshness, and context rules before admission.

**Blockchain-anchored trust.** The effectiveness of a proactive revocation decision depends solely on the infrastructure that logs and disseminates the decision. When revocation is stored in a local database, an attacker can easily manipulate the revocation history if a gateway is compromised. Additionally, the use of multiple gateways in various locations may cause the gateways to have differing opinions about which credentials are valid. This work therefore treats the ledger as a first-class architectural component, evaluated through a parameterized ledger model rather than as an executed blockchain deployment. Every accept, challenge, or revoke decision is condensed into compact evidence, batched into a Merkle tree [5], and anchored on a permissioned blockchain such as Hyperledger Fabric [6]; revocations are additionally propagated through an on-chain revocation-registry smart contract, and device identities are bound to W3C Decentralized Identifiers (DIDs) with verifiable credentials [7]. The result is a tamper-evident audit architecture for submitted evidence and a convergent published revocation-state model under the stated ledger assumptions, with O(1) logical on-chain payload per decision batch; it does not prove completeness for events suppressed before submission.

**Contribution.** This article adapts and extends the Yang–Baxter consistency methodology into an IoT- and blockchain-specific framework while preserving the same structural components: state of the art, cryptanalytic foundations, Yang–Baxter traversal, Brauer-algebra interpretation, worked example, Zero-Trust framework, decision algorithms, and performance-evaluation methodology. The contributions are:

- YB-IoT-CG, a Zero-Trust IoT post-authentication admission gateway that combines conventional authentication, redundant Yang–Baxter execution-consistency checking, deterministic freshness/context policy, and ledger-backed revocation, is proposed.
- An IoT credential transcript is defined that binds the device identifier, token, nonce, timestamp, firmware state, and telemetry digest.
- A Yang–Baxter-inspired redundant verification step is formalized for the restricted isolated single-path intermediate-state perturbation class of Proposition 7; it is not presented as a detector of arbitrary externally forged credentials.
- TMTO endpoint-proximity concepts are recharacterized for proactive credential revocation under constrained edge resources, while the Brauer construction is retained only as a structural/combinatorial descriptor and not as cryptographic entropy.
- A blockchain-backed architectural design is specified: Merkle-batched audit anchoring on a permissioned ledger, an on-chain revocation-registry smart contract, DID-based device identity binding, and a bounded-staleness synchronization protocol between edge caches and the ledger.
- Algorithms are provided for access decision, adaptive threshold recalibration, contextual risk governance, Merkle-batch anchoring, and on-chain revocation propagation.
- IoT- and blockchain-oriented evaluation metrics are defined for the composed post-authentication pipeline, while YB-specific evidence is restricted to the declared fault-injection class; reported latency is incremental post-authentication kernel time rather than end-to-end authentication latency.

The preceding seven contributions are retained. The revision additionally makes their formal scope explicit by (i) publishing the exact non-cryptographic reduction $\rho_{q}$ and its pigeonhole boundary; (ii) proving a domain-consistent affine Yang–Baxter family and the precise positive/negative consistency guarantees; (iii) defining bounded freshness, context, risk, and threshold-update functions; (iv) proving authentication-preservation, audit-integrity, revocation-monotonicity, conditional-staleness, and incremental-complexity properties; and (v) distinguishing modeled logical ledger quantities from live-ledger or hardware measurements.

**Paper organization.** The remainder of the article is organized as follows. Section 2 presents the related work. The threat model and assumptions are defined in Section 3. Section 4 introduces the mathematical foundation. Section 5 presents the YB-IoT-CG protocol, including the consistency scheme, the architecture, and the decision algorithms. Section 6 describes the blockchain-backed architectural design, including the smart-contract data model, Merkle anchoring, DID integration, and the cost model. Section 7 provides the security analysis. The Python-based experimental setup is described in Section 8. Section 9 presents and discusses the simulation results, including blockchain anchoring overhead. Section 10 discusses the limitations of this study, and Section 11 concludes the article.

## 2. Related Work

IoT authentication has evolved from static shared secrets to certificate-based architectures, lightweight public-key cryptography, device attestation, blockchain-assisted identity, and Zero-Trust access models [2]. In 6G-enabled IoT environments, this evolution has also been connected with access-control mechanisms for highly distributed devices [8]. The literature can be grouped into six directions.

**Lightweight authentication.** Resource-constrained devices motivate efficient schemes based on hash functions, elliptic curves, physically unclonable functions (PUFs), and symmetric-key protocols [9,10]. Healthcare-oriented authentication studies further emphasize lightweight and anonymity-preserving protection for constrained IoT users [11]. These approaches reduce computation, but many still assume that a key remains valid until explicit expiration or revocation.

**Certificate and PKI-based IoT identity.** Device certificates and mutual TLS offer strong identity binding [12]. However, certificate lifecycle management is difficult in low-cost IoT deployments. Revocation lists and online certificate status checks are often heavy, delayed, or unavailable when devices operate intermittently.

**Blockchain and oracle-based access control.** Blockchain-backed identity and smart-contract authorization improve auditability and non-repudiation [13]. Zero-knowledge mutual authentication strengthens privacy in this line [14]. Sidechain-based access and trust mechanisms extend blockchain control for IoT networks [15]. Decentralized identity management with DIDs and verifiable credentials has been demonstrated over IOTA for IoT devices [16], and permissioned platforms such as Hyperledger Fabric provide execute-order-validate architectures suited for consortium IoT deployments [6]. Oracle networks connect external physical data to ledgers. However, most existing stacks focus on identity verification, data correctness, or consensus, and much less on how administratively defined credential-risk indicators can be evaluated before admission, or how a deterministic, explainable pre-admission verdict can be anchored on-chain with bounded cost. No cryptanalytic meaning is assigned to geometric proximity in the reduced state space.

**Trusted execution and attestation.** TEEs can protect signing operations and generate attestation evidence. Nevertheless, TEEs do not automatically solve weak credential selection, token replay, or collision-prone key evolution. A compromised or stale credential may still be presented to a trusted enclave unless an upstream risk gate rejects it [8].

**Machine-learning anomaly detection.** ML-based IoT security detects abnormal traffic, device behavior, or telemetry patterns [17]. Such methods are useful but often operate after data has already entered the pipeline. They may also require training data and may produce opaque decisions.

**Algebraic and cryptanalytic consistency.** The proposed work complements these lines by adding a lightweight algebraic consistency check before credential use. Instead of waiting for behavioral anomalies, the gateway verifies whether the credential transcript respects a Yang–Baxter traversal and whether its digest is near a risky TMTO chain endpoint, and it commits the outcome to a permissioned blockchain for tamper-evident propagation.

**Algebraic and cryptanalytic consistency.** The broader Yang–Baxter literature is substantially larger than the IoT-authentication subset considered in the comparative table. Set-theoretic solutions, brace-based formulations, and related algebraic constructions establish structural properties such as bijectivity, involutivity, non-degeneracy, and braid consistency [3,18], while Brauer configuration algebras provide a separate combinatorial framework for describing recurrent algebraic structures [19]. These works are mathematically relevant to the foundation of YB-IoT-CG, but they are not direct IoT credential-authentication baselines. Conversely, blockchain-assisted IoT authentication and Zero-Trust access control are already established directions; this work does not claim novelty for that combination itself. Within the literature reviewed for this revision, we did not identify a scheme that uses equality of two Yang–Baxter traversals specifically as a deterministic post-authentication execution-consistency/traceability invariant over authenticated IoT credential metadata and then couples that invariant with freshness/context screening, proactive revocation, and permissioned-ledger audit propagation.

Ali and Al-Sharafi [17] and Basri et al. [20] are cited here before the comparative table because they motivate the two experimental simulator profiles discussed later. Their source papers report, respectively, Random-Forest accuracy of 94% (with LSTM 92%) and 98.9% attack-detection accuracy in the reported source setting; the separate 96.50% and 98.10% values used later are outputs of the common synthetic simulator and are not attributed to these publications.

**Comparative synthesis.** Table 1 summarizes representative recent contributions from IEEE and MDPI venues related to IoT authentication, blockchain-assisted access control, fog/edge security, Zero-Trust architectures, decentralized identity, and privacy-preserving credential management. The reviewed works show a clear trend toward distributed trust, blockchain-backed auditability, lightweight cryptography, attribute-based access control, zero-knowledge authentication, and quantum-resistant mechanisms. However, most of these approaches focus on identity verification, privacy preservation, access-policy enforcement, or post-event attack detection, and they typically use blockchains as passive audit stores. In contrast, YB-IoT-CG introduces a deterministic algebraic credential-screening layer that evaluates transcript consistency before telemetry admission, uses Yang–Baxter path divergence only as evidence of the declared internal execution inconsistency, while replay/clone/context decisions are explained by the corresponding freshness, context, and registry rules, and actively uses the blockchain as the authoritative substrate for revocation propagation and Merkle-anchored audit evidence with O(1) per-batch storage.

The blockchain-IoT approaches summarized in Table 1, including lightweight PoS-oriented designs, are architectural comparators rather than directly interchangeable packet-level decision kernels. Likewise, ECC, MAC, signature, and certificate mechanisms establish cryptographic authenticity; the proposed Yang–Baxter layer is complementary and post-authentication.

## 3. Threat Model and Assumptions

### 3.1. System Model

The system contains a set of IoT devices $D=\{D_{1},\ldots ,D_{N}\}$, one or more edge gateways *G*, a cloud or local application service *S*, a permissioned blockchain network L maintained by consortium peers, and an audit backend *A* that consumes ledger events. Each device periodically sends a message [1]. Fog-enabled IoT surveys motivate the same edge-to-cloud abstraction [27].

(1) $$M_{i}=\left(d_{i},\tau_{i},n_{i},t_{i},p_{i},\sigma_{i}\right),$$

where $d_{i}$ is the device identifier, $\tau_{i}$ is the session token, $n_{i}$ is the nonce, $t_{i}$ is the timestamp, $p_{i}$ is the telemetry payload or its digest, and $\sigma_{i}$ is a MAC or digital signature. Each device identifier $d_{i}$ is bound to a Decentralized Identifier registered on L [7].

The operational order is compositional. First, the gateway executes standard syntax checks and the lower-layer authentication verifier, denoted ${\mathrm{Auth}}_{0}\left(M_{i}\right)$, using the already provisioned secret, public key, certificate, or equivalent trust anchor [23]. Only messages that pass ${\mathrm{Auth}}_{0}$ enter the YB-IoT-CG screening layer. The gateway then constructs the canonical screening record

(2) $$C_{i}=\left(d_{i},\tau_{i},n_{i},t_{i},H\left(p_{i}\right),{\mathrm{fw}}_{i},{\mathrm{ctx}}_{i}\right),$$

where ${\mathrm{fw}}_{i}$ is firmware-state evidence and ${\mathrm{ctx}}_{i}$ contains trusted gateway-observed context. The output is

(3) Decision∈{Accept,Challenge,Revoke},

and every decision produces compact evidence that is queued for Merkle batching, while confirmed revocations are additionally submitted to the modeled on-chain registry (Section 6). Table 2 summarizes the mathematical notation.

The gateway verifies standard authentication first or in parallel, but it does not immediately forward the message [23]. The continuous-verification view is consistent with Zero-Trust IoT access control [8]. It constructs an algebraic transcript and runs YB-IoT-CG. The output is

(4) Decision∈{Accept,Challenge,Revoke},

and every decision produces compact evidence that is Merkle-batched and anchored on L, while revocations are additionally written to an on-chain revocation registry (Section 6).

In the normative execution order used by the revised algorithms, ${\mathrm{Auth}}_{0}$ is completed before any stateful YB/risk update. The original “first or in parallel” wording above is retained as architectural history, but the security claims and formal propositions refer to the authentication-first control flow of Algorithm 1. Ledger submission is asynchronous and modeled; no synchronous chain operation is required for packet admission. The associated threshold recalibration and contextual decision are given in Algorithms 2 and 3, respectively.

### 3.2. Adversary Model

The considered adversary may:

- clone a sensor identifier;
- replay a previously valid token;
- forge an intermediate credential field;
- inject stale nonces or timestamps;
- attempt to exploit hash truncation or reduction collisions;
- compromise a low-cost node and reuse its credential in another location;
- send high-rate authentication attempts to force gateway overload;
- compromise a single edge gateway and attempt to rewrite or suppress local revocation and audit records.

The assumption is not that the adversary can break the hash function directly or that they control a Byzantine quorum of permissioned blockchain peers. However, they can target operational credential lifecycle loopholes, including weak keys, token reuse, invalid transcript updates, collision-related reductions, and manipulation of security states in stored devices. Studies on remote authentication also indicate an attack vector of replay and misuse of credentials in fog environments and IoT [9]. The goal of a gateway is not to substitute but to complement the cryptographic authentication method with an additional pre-error-scope screen that is proactive, transparent, and demonstrated on ledger [12].

While these assumptions are conditional trust boundaries, they do not imply that the reality holds true and that attackers will never be able to breach them. The loss of authenticity or secrecy is inevitable if the lower hash/MAC/signature/certificate or secret-key layer has failed. Since ledger guarantees only hold true whenever the relevant configured permissioned ledger safety/liveness and authorization assumptions are met, evidence that never reaches the batch cannot be proved with the help of Merkle anchoring even if the gateway is compromised.

### 3.3. Security Goals

The scheme targets the original six security goals, augmented by an explicit layered-authentication preservation goal:

1. **Layered authentication preservation:** No message rejected by the lower secret-based authentication layer can be converted into an accepted message by YB-IoT-CG.
2. **Transcript consistency:** Forged intermediate states should fail the Yang–Baxter equality.
3. **Freshness:** Replayed or stale nonces should increase the risk score.
4. **Proactive revocation:** Credentials near risky chain endpoints should be challenged before use.
5. **Low overhead:** The gateway must operate within IoT latency and memory budgets, and on-chain interaction must be amortized.
6. **Auditability:** Every rejection or override must be reconstructable from compact signed and Merkle-anchored logs.
7. **Revocation integrity and conditional convergence:** Once a revocation is committed and delivered under the stated ledger assumptions, honest gateways converge monotonically to the published revocation state; this does not prevent a compromised gateway from suppressing an event before submission.

### 3.4. Assumptions and Scope

The proposed framework assumes that the adversary does not directly break the underlying cryptographic hash function, MAC, digital signature, certificate, or secure transport primitive [23], and that the permissioned blockchain L satisfies its standard safety and liveness guarantees under the configured ordering service and endorsement policy [6]. Lightweight blockchain-assisted authentication also motivates careful treatment of credential lifecycles [9]. The considered attack surface is the operational credential lifecycle, including cloned identifiers, stale tokens, replayed nonces, forged intermediate credential states, abnormal credential trajectories, collision-prone reductions, and single-gateway state tampering. Under these assumptions, the purpose of YB-IoT-CG is to detect whether a credential transcript is algebraically consistent, fresh, and operationally acceptable before telemetry is admitted into the IoT application pipeline, and to record the verdict in a tamper-evident form.

YB-IoT-CG is not intended to replace conventional cryptographic authentication, network intrusion detection, firmware attestation, or physical tamper protection [1]. Delay-trust mechanisms remain complementary for communication-level MitM detection [20]. Rather, it operates as an additional credential-hygiene and admission-control layer located at the edge gateway and backed by the ledger. The scope of this work is therefore limited to deterministic credential screening, proactive revocation, contextual risk assessment, blockchain-anchored auditability, and explainable rejection of inconsistent credential transcripts. Network-wide malware detection, semantic validation of payload content, and physical side-channel analysis are outside the direct scope of the proposed kernel, although they may be integrated as complementary layers in a complete Zero-Trust IoT deployment [8].

The single-gateway compromise assumption is also restricted. Merkle roots and ledger transactions protect the integrity and ordering of evidence that has actually been submitted; they do *not* prove completeness when a compromised gateway suppresses an event before submission. Signed receipts, monotone sequence numbers, cross-gateway replication, and remote attestation are appropriate hardening mechanisms for a production deployment, but they are outside the present simulation. Consequently, the manuscript no longer claims that a single compromised gateway can never hide an unsubmitted local event.

Finally, the current 1000-packet/30% attack experiments are controlled packet-level simulations and are not a large-scale DoS or revocation-storm benchmark. Ordering backpressure, network partitions, missed ledger events, cache reconnection/resynchronization, hardware energy consumption, and high-rate distributed flooding are explicitly treated as limitations in Section 10.

## 4. Mathematical Foundation

### 4.1. Canonical Credential Digest and Exact Reduction

A single normative digest definition is used throughout the protocol, pseudocode, simulation, audit records, and Merkle proofs. Let

(5) $$C_{i}=\left(d_{i},\tau_{i},n_{i},t_{i},H\left(p_{i}\right),{\mathrm{fw}}_{i},{\mathrm{ctx}}_{i}\right)$$

be the post-authentication screening record. Define ${\mathrm{enc}}_{C}$ as an injective, versioned type–length–value encoding. Its first field is the ASCII domain-separation string YB-IoT-CG/CRED/v1; every subsequent field is encoded as

(6) TLV(T,x)=T ‖ I2OSP(|enc(x)|,4) ‖ enc(x),

where *T* is a one-byte field/type tag, the four-byte length is unsigned big-endian, byte strings are copied verbatim, text identifiers are UTF-8 encoded, integers (including $t_{i}$) use a fixed unsigned 64-bit big-endian representation, $H(p_{i})$ is exactly 32 bytes, and structured firmware/context objects are serialized as ordered typed field sequences with lexicographically ordered field names. The tags are fixed as 01–07 for $\left(d_{i},\tau_{i},n_{i},t_{i},H\left(p_{i}\right),{\mathrm{fw}}_{i},{\mathrm{ctx}}_{i}\right)$, respectively. The canonical digest is therefore

(7) $$h_{i}=H\;\left({\mathrm{enc}}_{C}\left(C_{i}\right)\right).$$

No raw variable-length concatenation is normative. The full 256-bit $h_{i}$ is retained for audit/security binding; the algebraic reduction below is only an operational state and is never treated as a cryptographic authenticator.

Let $h_{i}$ be a 256-bit digest partitioned into four consecutive 64-bit words, and let OS2IP(·) convert an 8-byte string to a non-negative integer. The implementation uses the first three words:

(8) $$\begin{array}{cc} u_{i,0} & =\mathrm{OS2IP}(h_{i}\left[0:8\right]),\\ u_{i,1} & =\mathrm{OS2IP}(h_{i}\left[8:16\right]),\\ u_{i,2} & =\mathrm{OS2IP}(h_{i}\left[16:24\right]), \end{array}$$

and defines

(9) $$\rho_{q}\left(h_{i}\right)=\left(u_{i,0}\;\mathrm{mod}\;q,\;u_{i,1}\;\mathrm{mod}\;q,\;u_{i,2}\;\mathrm{mod}\;q\right)=\left(a_{i},b_{i},c_{i}\right)\in Z_{q}^{3}.$$

For the simulation, q=997, hence $|Z_{q}^{3}|\;=997^{3}=991,026,973$ and

(10) $$\log_{2}\left(997^{3}\right)\approx 29.884\;\mathrm{bits}.$$

This value is far below modern cryptographic security levels and is reported precisely to prevent a security-strength interpretation of $\rho_{q}$. The reduced triple is an operational state only; authenticity and collision resistance remain bound to $\sigma_{i}$, ${\mathrm{Auth}}_{0}$, and the full digest $h_{i}$.

If a 64-bit word *U* is uniform on $\left\{0,\ldots ,2^{64}-1\right\}$ and $U_{q}=U\;\mathrm{mod}\;q$, write $2^{64}=qk+r$ with 0≤r<q. Then each residue occurs either *k* or k+1 times. For q=997, r=961. Therefore the maximum difference between the probabilities of any two residues is exactly $2^{-64}$. This is the ordinary finite-word modulo bias; neither perfect uniformity nor independence of the three reduced coordinates is required as a security assumption [28,29].

**Proposition** **1** (Pigeonhole boundary of the reduced state)**.** *For any reduction $\rho_{q}:{\left\{0,1\right\}}^{256}\to Z_{q}^{3}$ with q=997, at least two distinct 256-bit digests map to the same reduced triple. Consequently, equality of reduced triples cannot imply equality of full digests and cannot be used as a cryptographic authentication criterion.*

**Proof.**  The domain contains $2^{256}$ elements whereas the codomain contains only $q^{3}=991,026,973\;<\;2^{30}$ elements. Since $2^{256}>q^{3}$, the pigeonhole principle implies that $\rho_{q}$ is not injective. Thus $\rho_{q}\left(h\right)=\rho_{q}\left(h^{\prime }\right)$ does not imply $h=h^{\prime }$. The protocol therefore retains and authenticates the full digest and uses the reduced state only for constant-size algebraic processing.    □

### 4.2. TMTO-Inspired Proactive Credential Risk

A TMTO chain follows Hellman’s time–memory trade-off model [4]. Rainbow-table refinements motivate differentiated reduction behavior [30]. It is written as

(11) $$\left(k_{0},k_{1},\ldots ,k_{t}\right),\;k_{j+1}=\rho_{q}\left(H\left(k_{j}\right)\right).$$

The gateway does not need to store all possible credentials. It stores compact chain endpoints and metadata describing risky regions. The online risk score is

(12) $$R_{i}=\alpha R_{\mathrm{chain}}\left(h_{i}\right)+\beta R_{\mathrm{fresh}}\left(n_{i},t_{i}\right)+\gamma R_{\mathrm{ctx}}\left(d_{i},{\mathrm{ctx}}_{i}\right),$$

where $R_{\mathrm{chain}}$ measures chain proximity, $R_{\mathrm{fresh}}$ measures nonce and timestamp anomalies, and $R_{\mathrm{ctx}}$ measures contextual inconsistency.

A TMTO chain follows Hellman’s time–memory trade-off intuition [4]; rainbow-table refinements motivate compact endpoint indexing [30]. In YB-IoT-CG this component is a deterministic operational-risk heuristic, not a learned predictor and not a password-cracking security claim. Let $E_{\mathrm{risk}}$ be a policy-maintained set of endpoint states obtained from predeclared risky trajectories in the simulator or administratively supplied production indicators.

For $x,y\in Z_{q}^{3}$, define the cyclic distance in one coordinate by

(13) $$\delta_{q}\left(x_{j},y_{j}\right)=\min\{|x_{j}-y_{j}\left|,\;q\;-\;\right|x_{j}-y_{j}\left|\right\},$$

and the normalized toroidal distance by

(14) $$d_{q}\left(x,y\right)=\frac{1}{3\lfloor q/2\rfloor }\sum_{j=1}^{3}\delta_{q}\left(x_{j},y_{j}\right).$$

Because $0\leq \delta_{q}\leq \left\lfloor q/2\right\rfloor$, $d_{q}\left(x,y\right)\in \left[0,1\right]$. Moreover, $\delta_{q}$ is the shortest-path metric on the cycle $Z_{q}$, and the normalized $\ell_{1}$ sum in Equation (14) is therefore a metric on $Z_{q}^{3}$.

To keep the edge cost bounded, risky endpoints are indexed through the deterministic bucket function

(15) $$b\left(h\right)=\mathrm{OS2IP}\;\left(H(\mathrm{YB}\text{-}\mathrm{IoT}\text{-}\mathrm{CG}/\mathrm{BUCKET}/v1\;\|\;h)[0:4]\right)\;\mathrm{mod}\;J.$$

The bucket queried for message *i* is $B\left(h_{i}\right)\subseteq E_{\mathrm{risk}}$ and is maintained with the deployment bound $|B(h_{i})|\leq K$. Endpoints must be constructed only from predeclared administrative indicators or a disjoint training/configuration corpus; test labels and test-packet outcomes are prohibited from endpoint construction. Endpoint density is reported as $|E_{\mathrm{risk}}|/q^{3}$. The chain-proximity score is

(16) $$R_{\mathrm{chain}}\left(h_{i}\right)=\left\{\begin{array}{cc} 1-\min_{e\in B(h_{i})}d_{q}\left(\rho_{q}\left(h_{i}\right),e\right), & B(h_{i})\neq \emptyset ,\\ 0, & B(h_{i})=\emptyset . \end{array}\right.$$

Hence $R_{\mathrm{chain}}\in \left[0,1\right]$ and larger values mean closer proximity to an administratively risky endpoint. This score is a policy heuristic only: no claim is made that toroidal distance after hashing and reduction predicts key recovery, credential compromise, or any cryptanalytic event. Sensitivity to *J*, *K*, endpoint density, weights, and thresholds must therefore be reported as policy sensitivity rather than cryptographic strength.

#### 4.2.1. Explicit Freshness Function

Let $T_{i}^{G}$ be the gateway receipt time, let $\delta_{\mathrm{clk}}\geq 0$ be the tolerated clock-skew bound, and define the effective age

(17) $$A_{i}=\max\left\{0,T_{i}^{G}-t_{i}-\delta_{\mathrm{clk}}\right\}.$$

Choose policy constants $0\leq \Delta_{\mathrm{soft}}<\Delta_{\mathrm{hard}}$. Define

(18) $$R_{\mathrm{age}}\left(i\right)={\mathrm{clip}}_{\left[0,1\right]}\;\left(\frac{A_{i}-\Delta_{\mathrm{soft}}}{\Delta_{\mathrm{hard}}-\Delta_{\mathrm{soft}}}\right),$$

where ${\mathrm{clip}}_{\left[0,1\right]}\left(x\right)=\min\left\{1,\max\left\{0,x\right\}\right\}$. Let $N_{d_{i}}\left(W\right)$ be the nonce set observed from *successfully authenticated* messages of device $d_{i}$ inside the active replay window *W*. Then

(19) $$R_{\mathrm{fresh}}\left(n_{i},t_{i}\right)=\max\left\{1\left[n_{i}\in N_{d_{i}}\left(W\right)\right],\;R_{\mathrm{age}}\left(i\right)\right\}.$$

Thus authenticated nonce reuse produces $R_{\mathrm{fresh}}=1$, while timestamp age increases continuously from zero to one between the soft and hard age limits. Unauthenticated traffic is excluded from $N_{d_{i}}\left(W\right)$, preventing an attacker from poisoning the freshness state by merely spoofing a victim identifier.

#### 4.2.2. Explicit Contextual Function

Let $\phi_{1},\ldots ,\phi_{m}\in \left[0,1\right]$ be normalized context-violation severities, for example firmware-policy mismatch, unexpected gateway class, location-class deviation, maintenance-state mismatch, or rate anomaly. Let $w_{\ell }\geq 0$ and $\sum_{\ell =1}^{m}w_{\ell }=1$. Define

(20) $$R_{\mathrm{ctx}}\left(d_{i},{\mathrm{ctx}}_{i}\right)=\sum_{\ell =1}^{m}w_{\ell }\phi_{\ell }\left(d_{i},{\mathrm{ctx}}_{i}\right).$$

This definition permits binary predicates ($\phi_{\ell }\in \left\{0,1\right\}$) or graded severities while keeping the score normalized. with α,β,γ≥0 and α+β+γ=1. The thresholds satisfy

(21) $$0\leq \theta_{\mathrm{risk}}<\theta_{\mathrm{revoke}}\leq 1.$$

The weights and thresholds are policy parameters fixed before a simulation run or deployment epoch; they are not learned from the evaluation labels.

**Proposition** **2** (Bounded risk score)**.** *Under Equations *(16)*, *(19)*, and *(20)*, the aggregate score in Equation *(12)* satisfies $0\leq R_{i}\leq 1$.*

**Proof.**  Each component belongs to [0,1]. Since α,β,γ are non-negative and sum to one, $R_{i}$ is a convex combination of three numbers in [0,1]. Therefore $R_{i}\in \left[0,1\right]$.    □

### 4.3. Brauer-Configuration Structural View with Explicit Assumptions

As in the original formulation, the chain graph can be interpreted as a quiver $Q_{\mathrm{IoT}}$. Each credential state is a vertex and each reduction step is a directed edge. The gateway groups recurrent credential trajectories as cycles and realizes them as a Brauer configuration [19] B. This interpretation is structural only: it is not used by the access-decision algorithm and contributes neither authentication nor cryptographic entropy.

For a Brauer configuration algebra $\Lambda_{B}$, the general dimension formula is

(22) $$\dim_{k}\Lambda_{B}=2\left|B_{1}\right|+\sum_{\alpha \in B_{0}}\mathrm{val}\left(\alpha \right)\left(\mu (\alpha )\mathrm{val}(\alpha )-1\right).$$

Let $m=|B_{1}|$ be the number of polygons/chain representatives and $n=|B_{0}|$ the number of configuration vertices. If every relevant vertex has multiplicity μ(α)=1 and valency val(α)=2, then

(23) $$\dim_{k}\Lambda_{B}=2m+2n.$$

Only under the additional incidence condition n=m/2 does the restricted expression reduce to 3m.

**Proposition** **3** (Restricted Brauer-configuration dimension)**.** *If $m=|B_{1}|$, $n=|B_{0}|$, and every configuration vertex has multiplicity one and valency two, then $\dim_{k}\Lambda_{B}=2m+2n$.*

**Proof.**  Substitution into Equation (22) makes every vertex contribute 2(2−1)=2, so the vertex sum is 2n and the polygon term is 2m.    □

For continuity with the original notation, the legacy scalar descriptor is retained as

(24) $$H_{B}=\log_{2}\;\left(\dim_{k}\Lambda_{B}\right),$$

but it is strictly a logarithmic structural-size index. It is not Shannon entropy, min-entropy, collision entropy, a cryptographic security parameter, or an input to the access-decision algorithm.

**Proposition** **4** (Reduced-state collision bound)**.** *Let $X_{1},\ldots ,X_{q_{o}}$ be independent reduced states in $Z_{q}^{3}$ with common distribution P. Define the collision probability of one draw by*

(25) $$\kappa_{P}=\sum_{x\in Z_{q}^{3}}P{\left(x\right)}^{2}.$$

*Then the probability of at least one pairwise collision among $q_{o}$ queries satisfies the union bound*

(26) Pr[collision]≤qo2κP.

*If, only for analytical illustration, the reduced states are modeled as uniform on $Z_{q}^{3}$, then $\kappa_{P}=1/q^{3}$ and*

(27) $$\mathrm{Pr}\left[\mathrm{collision}\right]\leq \min\;\left\{1,\frac{q_{o}\left(q_{o}-1\right)}{2q^{3}}\right\}.$$

**Proof.**  For every unordered pair (i,j), independence gives $\mathrm{Pr}\left[X_{i}=X_{j}\right]=\sum_{x}P{\left(x\right)}^{2}=\kappa_{P}$. Applying the union bound over the qo2 pairs gives Equation (26). The uniform special case follows from $P\left(x\right)=1/q^{3}$ [31].    □

The descriptor $H_{B}=\log_{2}\left(\dim_{k}\Lambda_{B}\right)$ is intentionally absent from this bound. No injective or probabilistic correspondence between Brauer-algebra dimension and sampled credential states has been established; consequently, $2^{\left\lfloor H_{B}\right\rfloor }$ is not used as an effective state-space cardinality. The only explicit state-space cardinality in the operational kernel is $q^{3}$, while the endpoint set $E_{\mathrm{risk}}$ remains an administrative policy object rather than a cryptographic collision space.

## 5. Proposed YB-IoT-CG Protocol

### 5.1. Pairwise Transformation

Let $X=Z_{q}$. A pairwise transformation is a map

(28) r:X×X→X×X.

For $X=Z_{q}$, write $r_{12}=r\times I_{X}$ and $r_{23}=I_{X}\times r$. A solution is involutive when $r^{2}=I_{X\times X}$ and non-degenerate when, writing $r\left(x,y\right)=(\sigma_{x}\left(y\right),\gamma_{y}\left(x\right))$, each component map is bijective [3,18]. The Yang–Baxter consistency condition is

(29) (r⊗I)(I⊗r)(r⊗I)=(I⊗r)(r⊗I)(I⊗r).

In IoT terms, the triple $\left(a_{i},b_{i},c_{i}\right)$ represents reduced values derived from device identity, session token, and nonce context. The two sides of (29) are two different checking orders over the same transcript.

### 5.2. A Practical Modular Map

For didactic and lightweight implementation, consider the map

(30) r(a,b)=(b+1,a−1)(mod10).

This map is easy to explain: the second value moves left and increases by one, while the first value moves right and decreases by one. As a didactic special case, this map is generalized below by the operational affine family; other production designs may also evaluate non-degenerate set-theoretic YBE solutions derived from a brace or finite-field construction [18].

The operational algebraic map is defined on the same domain used by the reduction $\rho_{q}$:

(31) $$r_{s}\left(x,y\right)=\left(y+s,\;x-s\right)\;\left(\mathrm{mod}\;q\right),\;s\in Z_{q}.$$

The simulation parameter q=997 and the lightweight instance s=1 are therefore mathematically consistent with the reduced credential state in Equation (9). The modulo-10 map above is retained as a didactic special case (q=10, s=1); the operational domain is generalized by the following result.

**Proposition** **5** (Affine involutive non-degenerate YBE solution)**.** *For every integer q≥2 and every $s\in Z_{q}$, the map $r_{s}$ of Equation *(31)* is bijective, involutive, non-degenerate, and satisfies the set-theoretic Yang–Baxter Equation *(29)*.*

**Proof.**  First,

(32) $$r_{s}\left(r_{s}\left(x,y\right)\right)=r_{s}\left(y+s,x-s\right)=\left(x-s+s,y+s-s\right)=\left(x,y\right)\;\left(\mathrm{mod}\;q\right),$$

so $r_{s}^{2}=I$; hence $r_{s}$ is bijective and its inverse is itself. Writing

(33) $$r_{s}\left(x,y\right)=\left(\sigma_{x}\left(y\right),\gamma_{y}\left(x\right)\right),\;\sigma_{x}\left(y\right)=y+s,\;\gamma_{y}\left(x\right)=x-s,$$

both component maps are translations of $Z_{q}$ and are therefore bijective, proving non-degeneracy.

It remains to verify the braid relation. For arbitrary $\left(a,b,c\right)\in Z_{q}^{3}$, the left composition gives

(34) $$\begin{array}{cc} \left(a,b,c\right) & \overset{r_{12}}{\to }\left(b+s,a-s,c\right)\\ & \overset{r_{23}}{\to }\left(b+s,c+s,a-2s\right)\\ & \overset{r_{12}}{\to }\left(c+2s,b,a-2s\right). \end{array}$$

The right composition gives

(35) $$\begin{array}{cc} \left(a,b,c\right) & \overset{r_{23}}{\to }\left(a,c+s,b-s\right)\\ & \overset{r_{12}}{\to }\left(c+2s,a-s,b-s\right)\\ & \overset{r_{23}}{\to }\left(c+2s,b,a-2s\right). \end{array}$$

The final triples are equal for every (a,b,c), so $r_{12}r_{23}r_{12}=r_{23}r_{12}r_{23}$ on $X^{3}$.    □

**Proposition** **6** (Closed form of the YB traversal)**.** *For the map $r_{s}$, both Yang–Baxter paths send every $\left(a,b,c\right)\in Z_{q}^{3}$ to*

(36) $$\Phi_{s}\left(a,b,c\right)=\left(c+2s,\;b,\;a-2s\right)\;\left(\mathrm{mod}\;q\right).$$

*Consequently, equality of the two honestly recomputed paths is an algebraic invariant and cannot, by itself, distinguish a forged external message from a legitimate external message.*

**Proof.**  The closed form follows directly from Equations (34) and (35). Since the equality holds for every input triple, replacing the external input by another triple and evaluating both paths correctly still yields equality. Authentication of the external message must therefore remain the responsibility of ${\mathrm{Auth}}_{0}$.    □

### 5.3. Valid Transcript Example

Let a reduced IoT credential be

(37) (a,b,c)=(1,2,3),

where 1 represents a reduced device identifier, 2 represents a reduced token, and 3 represents a reduced nonce.

The left traversal is

(38) $$\begin{array}{cc} \left(1,2,3\right) & \overset{r⊗I}{\to }\left(3,0,3\right)\\ \; & \overset{I⊗r}{\to }\left(3,4,9\right)\\ \; & \overset{r⊗I}{\to }\left(5,2,9\right). \end{array}$$

The right traversal is

(39) $$\begin{array}{cc} \left(1,2,3\right) & \overset{I⊗r}{\to }\left(1,4,1\right)\\ \; & \overset{r⊗I}{\to }\left(5,0,1\right)\\ \; & \overset{I⊗r}{\to }\left(5,2,9\right). \end{array}$$

Both paths end at (5,2,9); therefore, the transcript is consistent.

The preceding modulo-10 calculation is retained as the original didactic example. The same transcript in the operational simulation domain is: For the simulation domain q=997 and s=1, let

(40) (a,b,c)=(1,2,3).

The left traversal is

(41) $$\begin{array}{cc} \left(1,2,3\right) & \overset{r_{12}}{\to }\left(3,0,3\right)\\ \; & \overset{r_{23}}{\to }\left(3,4,996\right)\\ \; & \overset{r_{12}}{\to }\left(5,2,996\right), \end{array}$$

and the right traversal is

(42) $$\begin{array}{cc} \left(1,2,3\right) & \overset{r_{23}}{\to }\left(1,4,1\right)\\ \; & \overset{r_{12}}{\to }\left(5,0,1\right)\\ \; & \overset{r_{23}}{\to }\left(5,2,996\right). \end{array}$$

The common result agrees with Equation (36): (3+2,2,1−2)=(5,2,996)(mod997).

**Formal single-path fault model.** The useful security role of the double traversal is not external-message authentication but redundancy against a declared execution inconsistency. We therefore make the fault model explicit. Assume the right path is independently recomputed without fault. After the first left step, suppose the second coordinate is perturbed by $\varepsilon \in Z_{q}$:

(43) (b+s,a−s,c)⟶(b+s,a−s+ε,c).

**Proposition** **7** (Detection of a nonzero isolated intermediate perturbation)**.** *Under the fault model in Equation *(43)*, the final corrupted left path is*

(44) $${\tilde{L}}_{s}\left(a,b,c;\varepsilon \right)=\left(c+2s,b,a-2s+\varepsilon \right)\;\left(\mathrm{mod}\;q\right),$$

*whereas the independent right path is $R_{s}\left(a,b,c\right)=\Phi_{s}\left(a,b,c\right)$. Therefore*

(45) $${\tilde{L}}_{s}=R_{s}\;⟺\;\varepsilon \equiv 0\;\left(\mathrm{mod}\;q\right).$$

*Every nonzero perturbation in this declared fault class is detected by the path comparison.*

**Proof.**  Starting from the perturbed state,

(46) $$\begin{array}{cc} \left(b+s,a-s+\varepsilon ,c\right) & \overset{r_{23}}{\to }\left(b+s,c+s,a-2s+\varepsilon \right)\\ & \overset{r_{12}}{\to }\left(c+2s,b,a-2s+\varepsilon \right). \end{array}$$

By Proposition 6, the unperturbed right output is (c+2s,b,a−2s). The first two coordinates coincide, and the third coordinates coincide exactly when ε≡0(modq).    □

For the numerical example above, injecting ε=8 after the first left step changes (3,0,3) to (3,8,3). The corrupted left output becomes (5,2,7), whereas the independent right output remains (5,2,996); the mismatch is therefore detected.

The proposition is deliberately scoped. A common-mode fault that identically corrupts both paths, a fault that is algebraically canceled before comparison, or a compromise of the comparison logic itself is outside this guarantee. These cases motivate implementation diversity, protected memory, attestation, or duplicated execution on distinct trust domains in a production system.

### 5.4. Mechanism Attribution and Analytical Ablation

To isolate the actual contribution of the Yang–Baxter stage, define $C_{0}$ as the post-authentication configuration containing ${\mathrm{Auth}}_{0}$, revocation-cache lookup, freshness, context, and registry policy, and $C_{1}=C_{0}+\mathrm{YB}$. Proposition 6 implies that, for every externally supplied triplet that is correctly recomputed along both paths, the YB predicate in $C_{1}$ is always true. Therefore, in the absence of an internal execution fault, $C_{0}$ and $C_{1}$ produce identical security decisions for replay, clone, stale-message, and arbitrary external-field modification classes; the YB detection gain on those classes is exactly zero under the declared model. Their detection is attributed to ${\mathrm{Auth}}_{0}$, freshness, context, or the revocation registry.

The YB-specific positive contribution is evaluated only in the separate isolated fault-injection class of Proposition 7, for which $C_{0}$ has no YB comparator and $C_{1}$ detects every nonzero perturbation of the stated form. Structurally, the YB-only incremental work is exactly six evaluations of $r_{s}$ (three per path) plus one final triple comparison; no YB-only wall-clock latency is claimed because the reported timing instrument measures the complete post-authentication kernel. This analytical ablation prevents the aggregate 99.4% decision accuracy of the complete policy from being misreported as a Yang–Baxter detection rate. Likewise, the reported 7.8 μs is the complete incremental post-authentication kernel time and is not presented as the latency of the YB traversal alone.

**Scope of the ablation evidence.** The ablation reported above is analytical/model-level rather than an empirical with/without-YB experiment. The present study did not execute a separate randomized implementation-level fault-injection campaign. The simulator instruments the complete post-authentication kernel rather than an independently timed YB stage and does not implement a randomized internal execution-fault injector. Accordingly, no empirical YB-only wall-clock latency or implementation-level fault-detection rate is claimed. The quantitative YB-specific statements are limited to the zero detection gain for correctly recomputed external-message classes, the complete detection guarantee for the declared nonzero single-path perturbation class of Proposition 7, and the structural incremental cost of six evaluations of $r_{s}$ plus one final triple comparison. A dedicated empirical with/without-YB ablation and randomized fault-injection campaign is left for future work.

### 5.5. Forged Intermediate State

Suppose the adversary changes an intermediate value after the first left step:

(47) (3,0,3)⟶(3,8,3).

The forged continuation is

(48) $$\begin{array}{cc} \left(3,8,3\right) & \overset{I⊗r}{\to }\left(3,4,7\right)\\ & \overset{r⊗I}{\to }\left(5,2,7\right). \end{array}$$

The legitimate right path remains (5,2,9). Since

(49) (5,2,7)≠(5,2,9),

the gateway rejects the transcript. This example represents only an isolated internal perturbation of one execution path. It must not be interpreted as evidence that an externally modified token or credential field causes divergence when both paths are recomputed from the modified input.

**Figure 1:**
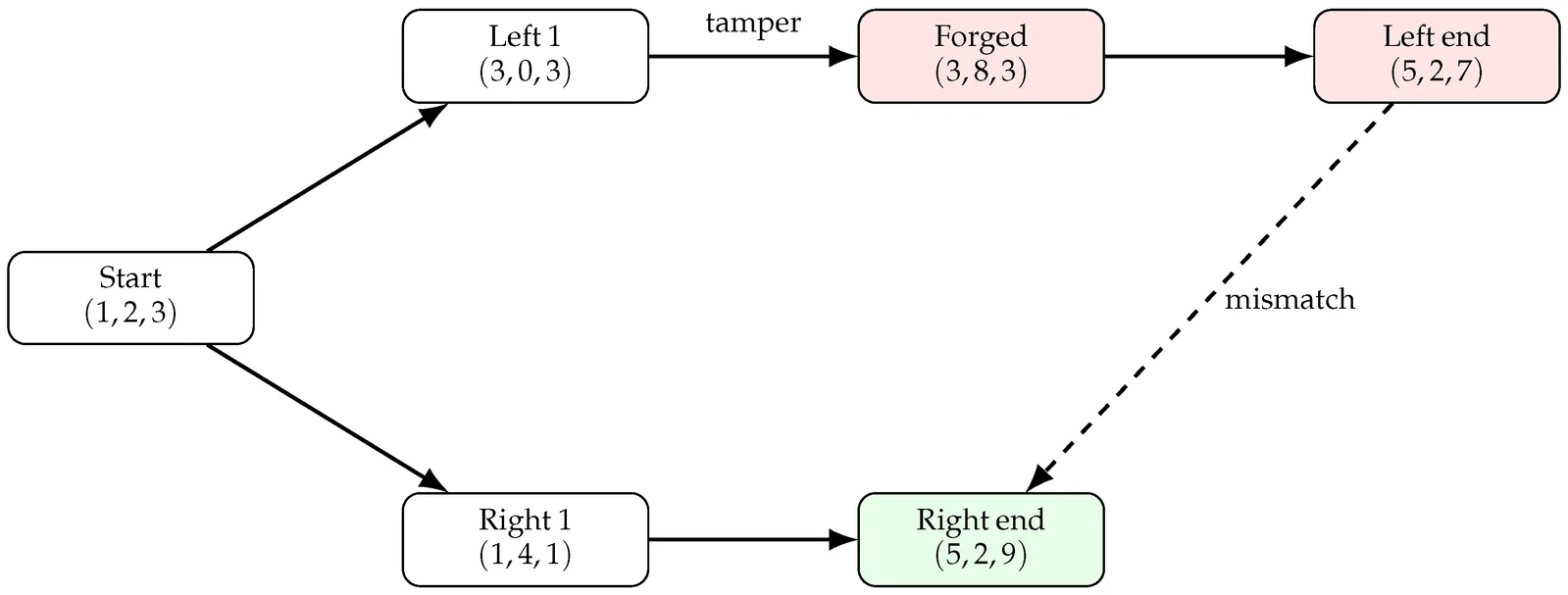
Explainable detection of the declared isolated single-path intermediate-state perturbation.

This original worked rejection is therefore interpreted only under the isolated intermediate-state perturbation model of Proposition 7. Proposition 6 shows that an arbitrary externally modified triple, if honestly recomputed along both paths, remains YB-consistent; external-message authenticity remains the responsibility of ${\mathrm{Auth}}_{0}$. Figure 1 illustrates the isolated intermediate-state perturbation.

### 5.6. Credential Classes

The gateway distinguishes three credential classes:

**Consistent and low-risk:** Both paths match and the risk score is below threshold. The message is accepted.
**Consistent but risky:** Both paths match, but TMTO proximity, context, or freshness is suspicious. The device is challenged.
**Inconsistent:** Traversal paths do not match. The message is challenged/quarantined as an execution-consistency failure; revocation requires independent corroborating evidence and the revocation threshold.

The third class is retained from the original taxonomy, but its irreversible-action semantics are narrowed: an isolated path inconsistency is challenged/quarantined by default, while on-chain revocation requires confirmed replay or corroborated multi-signal evidence together with $R_{i}\geq \theta_{\mathrm{revoke}}$, as specified by Algorithms 1 and 3.

### 5.7. YB-IoT-CG Architecture

**Figure 2:**
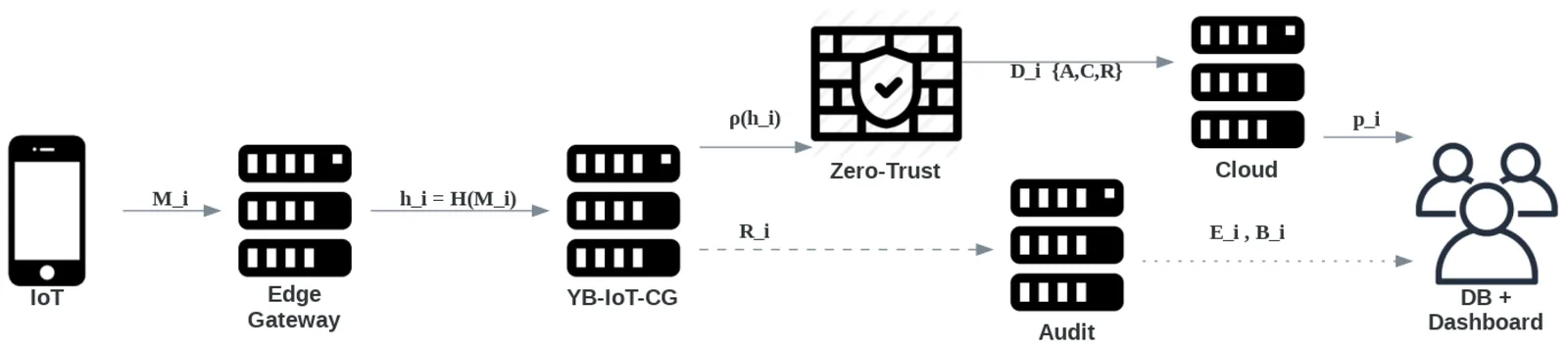
Operational architecture of the Yang–Baxter Internet of Things (IoT) Consistency Gateway (YB-IoT-CG) that is suggested. The architecture incorporates credential digest generation, Yang–Baxter consistency verification, TMTO-based risk assessment, Zero-Trust security implementation, secure communication of IoT devices, services made available in the cloud, telemetry persistence, and blockchain-provided audit ability and ability to revoke.

This proposed architecture keeps the original structure and conceptualization of the Layered Edge-to-Blockchain architecture as a continuous admission-control pipeline [32]. A Zero-Trust architecture evaluates this policy framework [2]. Figure 2 shows how an IoT message is processed in order to produce a credential digest, an algebraic transcript, a score via a TMTO-style indicator, and a decision (accept, challenge, or revoke) prior to the message reaching the cloud layer. All decisions are ultimately resolved via the permissioned ledger. The risk stage uses the TMTO idea proposed by Hellman [4] and refined in rainbow-table analysis [30].

### 5.8. Operational Architecture of YB-IoT-CG

Figure 2 illustrates the operational workflow of the proposed Yang–Baxter IoT Consistency Gateway (YB-IoT-CG). The architecture transforms conventional IoT authentication into a proactive credential-hygiene framework by integrating algebraic consistency verification, TMTO-inspired risk assessment, Zero-Trust enforcement, and blockchain-backed revocation and auditability within a unified edge-to-ledger security pipeline. Zero-Trust enforcement supports continuous verification [2]. TMTO analysis supports risk-aware credential screening [4]. Rainbow-table refinements inform compact endpoint monitoring [30]. Immutable evidence is consistent with classical timestamping and hash-linking mechanisms [33] and with Merkle authentication structures [5].

Unlike conventional authentication mechanisms that rely mainly on certificates, bearer tokens, or pre-shared keys, the proposed architecture introduces a mathematical verification layer before telemetry is admitted into the application domain [34]. Standard secure transport remains available through TLS [35]. IoT-specific TLS/DTLS profiles support constrained deployments [36]. This design is consistent with the threat model in Section 3: a credential may appear syntactically valid while still being stale, replayed, collision-prone, or contextually anomalous. Therefore, YB-IoT-CG treats authentication as a staged decision process rather than as a single binary check [2].

#### 5.8.1. IoT Device Layer

Each IoT node periodically emits a message

(50) $$M_{i}=\left(d_{i},\tau_{i},n_{i},t_{i},p_{i},\sigma_{i}\right),$$

where $d_{i}$ is the device identifier, $\tau_{i}$ is the session token, $n_{i}$ is the nonce, $t_{i}$ is the timestamp, $p_{i}$ is the telemetry payload or its digest, and $\sigma_{i}$ is a MAC or digital signature [34]. Constrained-device transport profiles motivate compact authentication exchanges [36]. This message is transmitted from sensors, PLCs, cameras, or smart meters toward the edge gateway. At this point, the telemetry is not yet trusted; it is only a candidate message that must pass both standard cryptographic checks and the proposed algebraic screening mechanism. The trust decision follows Zero-Trust reasoning [2]. Device capability baselines support the contextual interpretation of IoT evidence [37].

#### 5.8.2. Edge Gateway

The edge gateway acts as the first security boundary and normalizes the credential fields before cloud admission [32]. For every authenticated incoming message, the gateway constructs the canonical record $C_{i}$ and computes exactly the single normative digest $h_{i}=H\left({\mathrm{enc}}_{C}\left(C_{i}\right)\right)$ from Equation (7); no alternative concatenation is used in the architecture. The fields ${\mathrm{fw}}_{i}$ and ${\mathrm{ctx}}_{i}$ retain the meanings defined in Section 4 [37]. Before running the algebraic kernel, the gateway also consults its locally cached view of the on-chain revocation registry, so that credentials already revoked anywhere in the consortium are rejected immediately.

#### 5.8.3. Yang–Baxter Security Core

The core of the architecture maps the digest into an algebraic transcript:

(51) $$\rho_{q}\left(h_{i}\right)=\left(a_{i},b_{i},c_{i}\right)\in Z_{q}^{3}.$$

The transcript is then evaluated through the Yang–Baxter consistency condition [3]

(52) (r⊗I)(I⊗r)(r⊗I)=(I⊗r)(r⊗I)(I⊗r).

Operationally, the gateway computes two traversal outputs

(53) $$\begin{array}{c} L_{i}=\left(r⊗I\right)\left(I⊗r\right)\left(r⊗I\right)\left(a_{i},b_{i},c_{i}\right), \end{array}$$

(54) $$\begin{array}{c} R_{i}^{\mathrm{YB}}=\left(I⊗r\right)\left(r⊗I\right)\left(I⊗r\right)\left(a_{i},b_{i},c_{i}\right), \end{array}$$

and compares their final states. If $L_{i}=R_{i}^{\mathrm{YB}}$, the transcript is algebraically consistent. If $L_{i}\neq R_{i}^{\mathrm{YB}}$, the credential is immediately marked as inconsistent and the device is challenged, quarantined, or revoked depending on policy. This makes the rejection explainable because the gateway can report the exact pair of divergent traversal outputs.

#### 5.8.4. TMTO-Based Risk Assessment

A consistent transcript is necessary but not sufficient. A credential may satisfy the Yang–Baxter relation while still being operationally risky due to nonce reuse, chain proximity, abnormal context, or repeated credential trajectories [4]. Rainbow-table refinements motivate endpoint-proximity monitoring in compact chain structures [30]. For this reason, the security core computes the proactive score

(55) $$R_{i}=\alpha R_{\mathrm{chain}}\left(h_{i}\right)+\beta R_{\mathrm{fresh}}\left(n_{i},t_{i}\right)+\gamma R_{\mathrm{ctx}}\left(d_{i}\right),$$

where $R_{\mathrm{chain}}$ measures proximity to monitored TMTO chain endpoints, $R_{\mathrm{fresh}}$ evaluates nonce and timestamp freshness, and $R_{\mathrm{ctx}}$ captures contextual inconsistency. The weights α, β, and γ allow deployment-specific calibration. Thus, the gateway moves beyond reactive revocation and estimates whether a credential is approaching a risky operational region before the message is forwarded [4]. The differentiated-chain view is aligned with rainbow-table risk monitoring [30].

#### 5.8.5. Zero-Trust Enforcement Layer

The Zero-Trust firewall does not assume trust based on network location or prior enrollment [2]. Instead, it consumes the output of the security core and applies the decision rule

(56) $${\mathrm{Decision}}_{i}\in \left\{\mathrm{Accept},\mathrm{Challenge},\mathrm{Revoke}\right\}.$$

A low-risk and Yang–Baxter-consistent credential is accepted. A credential that is consistent but close to the risk threshold is challenged through additional evidence, such as device attestation, challenge-response, or re-authentication. An inconsistent or high-risk credential is revoked or quarantined before telemetry reaches the cloud platform, and the revocation is committed to the on-chain registry so that all consortium gateways converge to the same view. This realizes the Zero-Trust principle of continuous verification at the edge [2].

#### 5.8.6. Secure Communication Layer

Only accepted traffic is forwarded through standard IoT protocols such as MQTT, CoAP, TLS, or DTLS [38]. CoAP supports constrained application messaging [39]. TLS 1.3 provides the standard secure transport layer [35]. DTLS 1.3 extends transport protection to datagram settings [40]. IoT profiles adapt TLS and DTLS to constrained deployments [36]. The proposed architecture is therefore complementary to existing protocol stacks and does not require the replacement of deployed IoT communication mechanisms. YB-IoT-CG functions as an admission-control and credential-hygiene layer placed before application ingestion.

#### 5.8.7. Cloud Services and Telemetry Persistence

After authorization, telemetry is forwarded to cloud services, dashboards, or digital twins [41]. Edge computing motivates this gateway-to-cloud partitioning [32]. The cloud platform persists validated payloads in the telemetry database and makes them available for visualization, analytics, and operational decision making. Since the edge gateway filters suspicious credentials before ingestion, downstream services receive data streams that have already passed algebraic, cryptographic, contextual, and risk-based screening.

#### 5.8.8. Auditability and Blockchain Anchoring

Every decision is converted into compact audit evidence:

(57) $$E_{i}=\left(h_{i},R_{i},{\mathrm{Decision}}_{i}\right).$$

To preserve integrity and non-repudiation, the system aggregates evidence into Merkle batches and anchors

(58) $$B_{j}=\mathrm{MerkleRoot}\left(E_{jB+1},\ldots ,E_{(j+1)B}\right)$$

on the permissioned blockchain, where *B* is the batch size. Raw telemetry does not need to be written on-chain; only compact roots, revocation events, and metadata are anchored. This preserves privacy while enabling post-incident reconstruction. The full blockchain data model, smart contracts, and cost analysis are developed in Section 6.

#### 5.8.9. End-to-End Security Workflow

The complete workflow represented in Figure 2 is

(59) $$M_{i}\to h_{i}\to \rho_{q}\left(h_{i}\right)\to \mathrm{YBE}\to R_{i}\to {\mathrm{Decision}}_{i}\to \mathrm{Cloud}\to \mathrm{Blockchain}\;\mathrm{Audit}.$$

This chain connects the mathematical contribution of the article with the engineering deployment model. The architecture remains lightweight enough for edge gateways, explainable enough for security operators, verifiable through the ledger, and compatible with industrial, healthcare, environmental, and smart-city IoT deployments.

### 5.9. Relationship with the Five-Layer Framework and NIST Zero-Trust Tenets

The operational architecture in Figure 2 preserves the five conceptual layers proposed for YB-IoT-CG. Layer 1 corresponds to credential digest generation, transcript reduction, and Yang–Baxter checking. Layer 2 corresponds to sandboxed MAC, signature, certificate, or attestation verification. Layer 3 corresponds to risk governance through adaptive thresholds and contextual policies. Layer 4 corresponds to privacy-preserving telemetry forwarding, where raw payloads are minimized or masked before external persistence. Layer 5 corresponds to signed audit evidence, Merkle anchoring, and on-chain revocation over the permissioned blockchain. This mapping ensures that the simplified figure remains faithful to the complete security model while presenting the architecture in a form that is easier to evaluate, implement, and reproduce.

The architecture preserves the five conceptual functions of the original framework but changes their *operational order*: lower-layer MAC/signature/certificate verification is executed before stateful algebraic/risk processing. Conceptually, the functions are (i) cryptographic authentication and secure input normalization; (ii) credential digest/reduction and Yang–Baxter consistency; (iii) risk governance through bounded thresholds and contextual policy; (iv) privacy-preserving telemetry forwarding; and (v) signed audit evidence, Merkle anchoring, and revocation propagation.

The design also maps directly to the seven Zero-Trust tenets of NIST SP 800-207 [2]: (1) devices, gateways, ledgers, and telemetry services are treated as resources; (2) communications remain protected with TLS/DTLS regardless of network location; (3) admission is evaluated per message/session rather than inherited permanently; (4) decisions use dynamic policy over identity, freshness, firmware, and context; (5) device/firmware/context posture is continuously observed when available; (6) authentication and authorization are enforced before cloud admission and are re-evaluated after challenges; and (7) decision/audit telemetry is collected to support policy refinement and incident analysis. This mapping does not imply that YB-IoT-CG alone implements an entire enterprise Zero-Trust architecture; it identifies the specific tenets supported by this edge admission-control layer.

### 5.10. Decision Algorithms

The operational flow is deliberately conservative: inexpensive syntax/cache checks are followed by the pre-existing secret-based authentication layer; only authenticated messages reach stateful algebraic/risk processing; every branch emits audit evidence; and ambiguous or internally inconsistent evidence produces a challenge before irreversible revocation. The pseudocode is written so that the security invariants in Section 7 follow directly from control flow.

#### 5.10.1. Access Decision

The replay-history update is deliberately performed only after the freshness check and on every branch reached after successful ${\mathrm{Auth}}_{0}$ verification (Accept, Challenge, or corroborated Revoke). Thus the current nonce is not compared against itself, while Equation (19) and Algorithm 1 use the same definition of “successfully authenticated message.” The EWMA update applies only to $\theta_{\mathrm{risk}}$ and only after Accept; $\theta_{\mathrm{revoke}}$ remains fixed during the deployment epoch.
**Algorithm 1** YB-IoT-CG Layered Access Decision**Require:** IoT message $M_{i}=\left(d_{i},\tau_{i},n_{i},t_{i},p_{i},\sigma_{i}\right)$; ${\mathrm{fw}}_{i}$, ${\mathrm{ctx}}_{i}$**Require:** $0\leq \theta_{\mathrm{risk}}<\theta_{\mathrm{revoke}}\leq 1$; revocation cache $R_{\mathrm{cache}}$ 1: **if** ¬WellFormed$\left(M_{i}\right)$ **then** 2:       CommitAudit$\left(H\left(M_{i}\right),1,\mathrm{Challenge},\mathrm{Malformed}\right)$ 3:       **return** Challenge 4: **end if** 5: $\kappa_{i}\leftarrow H\left(\mathrm{DID}\left(d_{i}\right)\right)$ 6: **if**
 $\kappa_{i}\in R_{\mathrm{cache}}$ **then** 7:        CommitAudit$\left(H\left(M_{i}\right),1,\mathrm{Revoke},\mathrm{OnChainRevoked}\right)$ 8:        **return** Revoke▹ recognize existing revocation; do not create a new one 9: **end if**10: **if**
 ¬VerifyBaseAuth$\left(M_{i}\right)$ **then**11:        CommitAudit$\left(H\left(M_{i}\right),1,\mathrm{Challenge},\mathrm{InvalidBaseAuth}\right)$12:        **return** Challenge▹ unauthenticated traffic cannot update risk or enqueue revocation13: **end if**14: $C_{i}\leftarrow \left(d_{i},\tau_{i},n_{i},t_{i},H\left(p_{i}\right),{\mathrm{fw}}_{i},{\mathrm{ctx}}_{i}\right)$; $h_{i}\leftarrow H\left({\mathrm{enc}}_{C}\left(C_{i}\right)\right)$15: $\left(a_{i},b_{i},c_{i}\right)\leftarrow \rho_{q}\left(h_{i}\right)$16: $L\leftarrow r_{12}r_{23}r_{12}\left(a_{i},b_{i},c_{i}\right)$17: $R^{\mathrm{YB}}\leftarrow r_{23}r_{12}r_{23}\left(a_{i},b_{i},c_{i}\right)$18: **if**
 $L\neq R^{\mathrm{YB}}$ **then**19:        $e_{i}\leftarrow H(h_{i}\;\|\;L\;\|\;R^{\mathrm{YB}}\;\|\;\mathrm{YBE}\_\mathrm{Mismatch})$20:        CommitAudit$\left(h_{i},1,\mathrm{Challenge},e_{i}\right)$21:        RecordAuthenticatedNonce$\left(d_{i},n_{i},t_{i}\right)$22:        **return** Challenge23: **end if**24: *S*_fresh_ ← FreshnessScore$\left(n_{i},t_{i},d_{i}\right)$ using Equation (19)25: *S*_chain_ ← TMTOProximity$\left(h_{i},d_{i}\right)$ using Equation (16)26: *S*_ctx_ ← ContextScore$\left(d_{i},{\mathrm{ctx}}_{i}\right)$ using Equation (20)27: $R_{i}\leftarrow \alpha s_{\mathrm{chain}}+\beta s_{\mathrm{fresh}}+\gamma s_{\mathrm{ctx}}$28: D*_i_* ← ContextDECISION$\left(d_{i},R_{i},{\mathrm{ctx}}_{i}\right)$▹ Algorithm 329: **if**
 $D_{i}=\mathrm{Revoke}$ **then**30:        **if** $R_{i}\geq \theta_{\mathrm{revoke}}$ **then**31:              $e_{i}\leftarrow H(h_{i}\;\|\;R_{i}\;\|\;\mathrm{CorroboratedRisk})$32:              CommitAudit$\left(h_{i},R_{i},\mathrm{Revoke},e_{i}\right)$33:              QueueRevocation$\left(\kappa_{i},e_{i}\right)$34:              RecordAuthenticatedNonce$\left(d_{i},n_{i},t_{i}\right)$35:              **return** Revoke36:        **else**37:              CommitAudit$\left(h_{i},R_{i},\mathrm{Challenge},\mathrm{CorroborationBelowThreshold}\right)$38:              RecordAuthenticatedNonce$\left(d_{i},n_{i},t_{i}\right)$39:              **return** Challenge40:        **end if**41: **else if**
 $R_{i}\geq \theta_{\mathrm{risk}}$ **or**
 $D_{i}=\mathrm{Challenge}$ **then**42:        CommitAudit$\left(h_{i},R_{i},\mathrm{Challenge},\mathrm{PolicyRisk}\right)$43:        RecordAuthenticatedNonce$\left(d_{i},n_{i},t_{i}\right)$44:        **return** Challenge45: **end if**46: CommitAudit
 $\left(h_{i},R_{i},\mathrm{Accept},\mathrm{OK}\right)$47: RecordAuthenticatedNonce
 $\left(d_{i},n_{i},t_{i}\right)$48: $\theta_{\mathrm{risk}}\leftarrow BoundedEWMA\left(\theta_{\mathrm{risk}},R_{i},\lambda ,\eta ,\theta_{\min},\theta_{\max}\right)$▹ Algorithm 2; accepted traffic only49: **return** Accept

#### 5.10.2. Bounded Adaptive Threshold Recalibration

Algorithm 2 retains the EWMA idea but prevents unauthenticated, challenged, or revoked traffic from directly moving the threshold and limits the maximum update per accepted observation.
**Algorithm 2** Bounded EWMA Threshold Recalibration**Require:** $\theta_{t}\in \left[\theta_{\min},\theta_{\max}\right]$, $R_{i}\in \left[0,1\right]$ from an authenticated accepted message, λ∈[0,1], step bound η≥01:$\hat{\theta }\leftarrow \lambda R_{i}+\left(1-\lambda \right)\theta_{t}$2:$\hat{\theta }\leftarrow \min\left(\theta_{\max},\max\left(\theta_{\min},\hat{\theta }\right)\right)$3:$\theta_{t+1}\leftarrow \min\left(\theta_{t}+\eta ,\max\left(\theta_{t}-\eta ,\hat{\theta }\right)\right)$4:EmitMetric $\left(\theta_{t+1}\right)$5:**return** $\theta_{t+1}$

**Proposition** **8** (Bounded threshold invariant)**.** *Assume $0\leq \theta_{\min}\leq \theta_{t}\leq \theta_{\max}\leq 1$, $R_{i}\in \left[0,1\right]$, λ∈[0,1], and η≥0. Algorithm 2 satisfies*

(60) $$\theta_{t+1}\in \left[\theta_{\min},\theta_{\max}\right],\;\left|\theta_{t+1}-\theta_{t}\right|\leq \eta .$$

**Proof.**  The first clipping step places $\hat{\theta }$ in $\left[\theta_{\min},\theta_{\max}\right]$. The second step projects $\hat{\theta }$ onto the interval $\left[\theta_{t}-\eta ,\theta_{t}+\eta \right]$. Since that projection is taken toward a value already inside $\left[\theta_{\min},\theta_{\max}\right]$ and $\theta_{t}$ is also inside that interval, the result remains within the global bounds. By construction it also lies within η of $\theta_{t}$.    □

The proposition limits per-update drift; it does not by itself prove resistance to a long-horizon adaptive poisoning strategy. Such poisoning remains an experimental limitation.

#### 5.10.3. Contextual Challenge-Before-Revoke Logic

For reproducibility, let $\xi_{\ell }\in \left(0,1\right]$ be a policy severity threshold for context predicate $\phi_{\ell }$. Define the corroboration count

(61) $$N_{\mathrm{anom}}\left(i\right)=\sum_{\ell =1}^{m}1\left[\phi_{\ell }\left(d_{i},{\mathrm{ctx}}_{i}\right)\geq \xi_{\ell }\right],$$

and set multiSignalAnomaly=true only when $N_{\mathrm{anom}}\left(i\right)\geq 2$. Likewise, confirmedReplay=true requires authenticated nonce reuse plus session/device evidence defined by deployment policy; a mere unauthenticated reuse attempt is insufficient. Algorithm 3 summarizes the contextual decision logic.

Here confirmedReplay is asserted only from state derived from successfully authenticated traffic (for example, authenticated nonce reuse within the active window plus matching session/device policy evidence). Thus a spoofed unauthenticated packet cannot manufacture the corroborating condition used to enqueue a new on-chain revocation.
**Algorithm 3** Contextual Challenge or Corroborated Revocation**Require:** authenticated device $d_{i}$, risk $R_{i}$, context vector ${\mathrm{ctx}}_{i}$  1:  **if**
 ${\mathrm{ctx}}_{i}.\mathrm{firmwareUpdate}=\mathrm{true}$ **and**
 $\theta_{\mathrm{risk}}\leq R_{i}<\theta_{\mathrm{revoke}}$ **then**  2:         **return** Challenge  3: **else if** ${\mathrm{ctx}}_{i}.\mathrm{location}$ differs from registered class **then**  4:         **return** Challenge  5: **else if**
 ${\mathrm{ctx}}_{i}.\mathrm{nonceReuse}=\mathrm{true}$ **and**
 ${\mathrm{ctx}}_{i}.\mathrm{confirmedReplay}=\mathrm{false}$ **then**  6:         **return** Challenge  7: **else if**
 ${\mathrm{ctx}}_{i}.\mathrm{confirmedReplay}=\mathrm{true}$ **or**
 ${\mathrm{ctx}}_{i}.\mathrm{multiSignalAnomaly}=\mathrm{true}$ **then**  8:         **return** Revoke  9: **else if** ${\mathrm{ctx}}_{i}.\mathrm{burstRate}$ exceeds policy **then**10:         **return** Challenge11: **else**12:         **return** Accept13: **end if**

## 6. Blockchain-Backed Architectural Design of YB-IoT-CG

This section specifies the *proposed* ledger-facing architecture used by YB-IoT-CG. The present article does not claim an executed Hyperledger Fabric chaincode or EVM deployment. Instead, the data structures, pseudocode interfaces, Merkle batching, and revocation synchronization are evaluated through the parameterized ledger model described in Section 8. The design goals are (i) tamper-evident audit records for submitted evidence; (ii) convergent published revocation state across honest gateways; (iii) O(1) logical on-chain payload per decision batch; and (iv) strict decoupling between the low-latency edge decision path and the seconds-scale ledger path.

### 6.1. Ledger Selection and Deployment Topology

YB-IoT-CG targets consortium IoT deployments in which the participating organizations (plant operators, utilities, hospitals, municipalities) are identified but mutually semi-trusting. A permissioned platform such as Hyperledger Fabric is therefore the reference deployment target: its execute-order-validate architecture, channel-based data isolation, pluggable endorsement policies, and membership service provider fit the identified-participant setting and avoid the probabilistic finality and transaction fees of public proof-of-work networks [6]. Nevertheless, the on-chain data model defined below is deliberately minimal (fixed-size roots, status flags, and event logs), so it can also be realized as an EVM smart contract on a public or consortium Ethereum-family network when cross-organization neutrality is required [42].

Each edge gateway runs a lightweight ledger client. Ordering and endorsement peers are hosted by the consortium members in the fog or cloud tier; no constrained IoT device interacts with the blockchain directly. This topology keeps device-side cost at zero and confines ledger interaction to gateways, which possess the computing and connectivity resources required for transaction signing and event subscription.

The topology above is an architectural target rather than evidence of a live deployment. In the present study, peers/orderers, commit latency, and event delivery are represented by the parameterized ledger model; no constrained IoT node performs a blockchain operation directly.

### 6.2. On-Chain Data Model and Smart Contracts

Two smart contracts (chaincode in Fabric terminology) define the on-chain state:

1. **RevocationRegistry.** A key–value mapping from the DID of a device [7] to a status record $\left(\mathrm{status},h_{\mathrm{evidence}},t_{\mathrm{rev}},g_{\mathrm{issuer}}\right)$, where status∈{Active,Challenged,Revoked}, $h_{\mathrm{evidence}}$ is the digest of the divergent Yang–Baxter traversal pair or risk report, $t_{\mathrm{rev}}$ is the ledger timestamp, and $g_{\mathrm{issuer}}$ identifies the revoking gateway. State transitions are monotone under policy: Revoked can only be lifted through an explicit multi-party re-enrollment transaction endorsed by a quorum of organizations, which prevents a single compromised gateway from silently reinstating a revoked credential.
2. **AuditAnchor.** An append-only log of Merkle roots $B_{j}$ computed over batches of decision evidence (Equation (58)), following the classical Merkle authentication-tree construction [5] and hash-linked timestamping [33]. Each anchor transaction stores the root, the batch cardinality, the covering time window, and the gateway signature.

Listing 1 sketches the registry interface in a Solidity-style pseudocode; the Fabric realization exposes the same interface through chaincode functions with organization-level endorsement policies.

The listing is a proposed logical interface only. It is not reported as executed Fabric chaincode or as a deployed EVM contract, and the experimental section does not attribute live-chain measurements to it.

**Listing 1.** Revocation-registry smart-contract interface (pseudocode).
contract RevocationRegistry {

 enum Status { Active, Challenged, Revoked }

 struct Record {

  Status  status;

  bytes32 evidenceDigest; // H(L_i || R_i^YB || R_i)

  uint64  revokedAt;

  address issuerGateway;

 }

 mapping(bytes32 => Record) registry; // key: H(DID)
 
 event Revoked(bytes32 indexed did, bytes32 evidence);

 event Anchored(bytes32 indexed root, uint32 batchSize);
 
 function revoke(bytes32 did, bytes32 evidence) onlyGateway { ... }

 function challenge(bytes32 did, bytes32 evidence) onlyGateway { ... }

 function reinstate(bytes32 did) onlyQuorum { ... }

 function anchor(bytes32 merkleRoot, uint32 n) onlyGateway { ... }

 function statusOf(bytes32 did) view returns (Status) { ... }

}


### 6.3. Merkle-Batched Audit Anchoring

Writing one transaction per packet decision would be prohibitive at IoT message rates. Instead, evidence records are accumulated into batches of size *B* and condensed into a single Merkle root before submission (Algorithm 4). Any individual decision $E_{i}$ can later be proven to belong to an anchored batch with a logarithmic-size inclusion proof of $\left\lceil \log_{2}B\right\rceil$ sibling hashes [5], which is sufficient for dispute resolution and regulatory audit without storing raw evidence on-chain.

Writing one transaction per packet decision is avoided by accumulating evidence into batches of size *B*. Let enc(·) be an injective canonical serialization of a decision-evidence tuple and define the Merkle leaf

(62) $$\ell_{i}=H\;\left(\mathrm{enc}(h_{i},R_{i},{\mathrm{Decision}}_{i},{\mathrm{reason}}_{i})\right).$$

Internal nodes are computed as $H(\mathrm{enc}\left(v_{L},v_{R}\right))$ with explicit left/right ordering. Any decision can later be associated with an anchored root using an O(logB)-hash inclusion proof, while raw telemetry remains off-chain [5].

**Proposition** **9** (Merkle tamper evidence under collision resistance)**.** *Assume enc is injective and H is collision resistant. After a Merkle root is committed, replacing an included evidence tuple $E_{i}$ by a distinct tuple $E_{i}^{\prime }\neq E_{i}$ while keeping the same authenticated root and a valid inclusion path is computationally infeasible except by finding a collision in H (or breaking the signature/ledger mechanism that authenticates the root).*

**Proof.**  Because enc is injective, $E_{i}^{\prime }\neq E_{i}$ implies distinct leaf inputs. If their leaf hashes differ, a verifier recomputing the path obtains a different value at the first ancestor where the changed hash is used unless some later pair of distinct node inputs hashes to the same output. Thus a successful substitution with the same root yields a collision at the leaf or at an internal node. If instead the attacker replaces the committed root, it must also defeat the mechanism that authenticates and orders the root on the permissioned ledger.    □

**Algorithm 4** Merkle-Batch Audit Anchoring**Require:** evidence queue Q, batch size *B*, flush interval $T_{\mathrm{flush}}$1: **while** gateway is running **do**2:       collect $\ell_{i}=H\left(\mathrm{enc}\left(h_{i},R_{i},{\mathrm{Decision}}_{i},{\mathrm{reason}}_{i}\right)\right)$ into buffer E3:        **if** |E|≥B **or** elapsed $\geq T_{\mathrm{flush}}$ **then**4:              root←
MerkleRoot(E)5:              tx←
Sign$n_{sk_{G}}(\mathrm{anchor},\mathrm{root},|E|,\left[t_{\mathrm{start}},t_{\mathrm{end}}\right])$6:              SubmitAsync(L,tx)▹ non-blocking; edge path never waits7:              persist E and proofs off-chain; E←∅8:        **end if**9: **end while**


### 6.4. On-Chain Revocation Propagation, Monotonicity, and Conditional Staleness

Revocations are latency-critical and are therefore submitted individually and immediately rather than batched (Algorithm 5). Every gateway subscribes to Revoked events and maintains a local cache $R_{\mathrm{cache}}$ consulted at line 1 of Algorithm 1, so registry lookups never require a synchronous ledger round-trip on the packet path.
**Algorithm 5** On-Chain Revocation Propagation**Require:** revocation queue entry $\left(\kappa_{i},e_{i}\right)$ produced by Algorithm 1; ledger client for L 1: tx←
Sign*_sk__G_*$\left(\mathrm{revoke},\kappa_{i},e_{i}\right)$ 2: Submit
(L,tx)     **On event** Revoked(κ,e) **at any gateway:** 3: $R_{\mathrm{cache}}\leftarrow R_{\mathrm{cache}}\cup \left\{\kappa \right\}$ 4: EmitMetric (revocationLatency)


The revocation worker does not recompute the evidence digest and does not reconstruct the DID hash. The signed transaction contains exactly the $\left(\kappa_{i},e_{i}\right)$ pair queued by Algorithm 1, eliminating the previous interface/data-flow mismatch.

Within one enrollment epoch, order the registry states by

(63) Active≺Challenged≺Revoked.

An explicit quorum-authorized re-enrollment/reinstatement terminates the old epoch and creates a new credential epoch; it is not treated as a downward transition inside the same epoch.

**Proposition** **10** (Monotone revocation within an enrollment epoch)**.** *Assume the registry contract accepts only transitions that are non-decreasing under Equation *(63)*. Then, within one enrollment epoch, once a DID hash κ reaches Revoked, no later valid transaction in that epoch can return it to Active or Challenged.*

**Proof.**  Revoked is the maximum element of the finite order in Equation (63). A non-decreasing transition from the maximum can only remain at the maximum. Therefore all accepted subsequent transitions preserve Revoked until a separately authorized new enrollment epoch is created.    □

**Proposition** **11** (Conditional revocation staleness)**.** *Assume a synchronous interval in which $t_{c}^{\max}$ is a hard upper bound on ledger commit/finality time and $t_{e}^{\max}$ is a hard upper bound on event delivery to every honest subscribing gateway, with no network partition, missed event, or subscriber failure. Then the stale-use interval after revocation submission satisfies*

(64) $$\Delta_{\mathrm{stale}}\leq t_{c}^{\max}+t_{e}^{\max}.$$

*After this interval, every honest gateway that applies Algorithm 5 contains $\kappa_{i}$ in its revocation cache.*

**Proof.**  Let submission occur at time $t_{0}$. By assumption, the transaction is final no later than $t_{0}+t_{c}^{\max}$. The corresponding revocation event reaches every honest subscriber no later than an additional $t_{e}^{\max}$, hence by time $t_{0}+t_{c}^{\max}+t_{e}^{\max}$. Upon receipt, Algorithm 5 performs the set union $R_{\mathrm{cache}}\leftarrow R_{\mathrm{cache}}\cup \left\{\kappa_{i}\right\}$. Set union is idempotent and monotone, so repeated events cannot remove the entry. Algorithm 1 checks this cache before the YB/risk stage, establishing the stated bound under the premises.    □

If commit and propagation delays are modeled as random variables, as in the present simulation, Equation (64) is not an unconditional deterministic bound. The experiment therefore reports empirical quantiles/medians of $T_{c}+T_{e}$. Partitions, missed events, compromised subscribers, or contract-level authorization failures violate the proposition premises and require resynchronization/fail-close policies in a production system.

### 6.5. DID-Based Device Identity, Verifiable Credentials, and Storage Boundary

Device enrollment binds each physical device to a DID document anchored on L, containing the device public key, its organizational controller, and service endpoints [7]. Session tokens $\tau_{i}$ are issued as short-lived verifiable credentials signed by the organizational issuer, so that the field $d_{i}$ entering the digest of Equation (7) is a ledger-resolvable identifier rather than an opaque string. This closes the loop between the algebraic kernel and the ledger: the same identifier that is screened by the Yang–Baxter transcript is the key of the on-chain revocation record, which prevents identifier aliasing between the screening and revocation layers. Prior IoT DID deployments demonstrate the feasibility of this lifecycle on constrained hardware [16].

W3C DID Core does not prescribe a fixed byte size because documents and representations are variable. In the proposed constrained-device topology, the full DID document is *not* stored on every IoT node. The device needs only its local key/secure-element reference, its compact identifier/token material, and the information required by the lower authentication protocol; DID resolution, full document metadata, and revocation state are handled by the gateway/ledger tier. This design avoids imposing JSON/JSON-LD document storage on constrained sensors while preserving a common ledger-resolvable identity key.

### 6.6. Cost, Latency, Verifiability, and Batch-Size Trade-Off

Let $t_{\mathrm{YB}}$ denote the per-packet kernel time (Equation (74)), $t_{\mathrm{tx}}$ the ledger commit latency, and *B* the anchoring batch size. Because anchoring is asynchronous, the per-packet decision delay remains $t_{\mathrm{YB}}$; the amortized ledger overhead per decision is

(65) $$T_{\mathrm{anchor}}=\frac{t_{\mathrm{tx}}}{B},\;S_{\mathrm{chain}}=\frac{|B_{j}|\;+\;\mathrm{meta}}{B}\in O\left(1/B\right)$$

bytes of on-chain storage per decision, where $|B_{j}|\;=\;32$ bytes for a 256-bit root. For B=64 and a Fabric commit latency of approximately two seconds, the amortized anchoring cost is on the order of tens of milliseconds per decision and under one byte of ledger storage per packet, while each revocation costs one dedicated transaction plus the staleness bound of Proposition 11. On EVM networks the same O(1)-per-batch property holds, with cost dominated by a single SSTORE and event emission per anchor [42]. This model is validated empirically in Section 9.2.

(66) $$0\leq T_{\mathrm{fill}}\leq \min\left(\frac{B-1}{\lambda_{d}},T_{\mathrm{flush}}\right),\;T_{\mathrm{verify}}=T_{\mathrm{fill}}+t_{\mathrm{tx}}.$$

If a record is approximately uniformly positioned inside a size-*B* batch and the count trigger occurs before the timer, the familiar mean waiting approximation is

(67) $$E\left[T_{\mathrm{fill}}\right]\approx \frac{B-1}{2\lambda_{d}}.$$

With an active timeout, the actual expectation depends on the arrival process and timer/reset policy; it is therefore not claimed to equal the minimum of two independent expectations. Physical ledger cost additionally includes transaction envelopes, signatures, indexing, block headers, endorsement metadata, and peer replication and is not measured by the present logical model.

For the nominal simulation rate of 100 decisions/s and $t_{\mathrm{tx}}=2$ s, the analytical sensitivity in Table 3 explains the choice B=64: smaller batches reduce fill delay but increase transaction frequency and logical payload per decision; larger batches improve amortization but increase waiting time before an individual audit record becomes anchored.

The table is an analytical sensitivity study, not a new Fabric benchmark. It is included to make the batch-size trade-off explicit without expanding the present work beyond its simulation scope.

## 7. Security Analysis

This section analyzes the security properties provided by the proposed YB-IoT-CG protocol. The analysis does not claim to replace the security guarantees of the underlying cryptographic primitives. Instead, it shows how the Yang–Baxter credential kernel adds a deterministic consistency layer for detecting forged, replayed, cloned, stale, or operationally risky credential transcripts before telemetry is admitted into the IoT application pipeline, and how the blockchain layer protects the integrity and propagation of the resulting verdicts.

**Formal compositional properties.** The formal claims below are compositional. ${\mathrm{Auth}}_{0}$ supplies cryptographic authenticity; the Yang–Baxter stage supplies a deterministic execution-consistency invariant under the declared fault model; the policy layer supplies bounded freshness/context governance; and the ledger layer supplies tamper evidence and revocation-state dissemination under its stated assumptions.

**Proposition** **12** (No authentication bypass)**.** *Assume Algorithm 1 is executed as specified and the only transition into the post-authentication YB/risk stage occurs after *VerifyBaseAuth*$(M_{i})=\mathrm{true}$. Then an external message for which ${\mathrm{Auth}}_{0}\left(M_{i}\right)=0$ cannot reach the Accept branch. Hence the probability of unauthenticated acceptance by the composed system is no greater than the false-acceptance/forgery probability of ${\mathrm{Auth}}_{0}$ plus implementation-failure probabilities outside the algorithmic model.*

**Proof.**  If ${\mathrm{Auth}}_{0}\left(M_{i}\right)=0$, line VerifyBaseAuth of Algorithm 1 takes the failure branch and returns Challenge immediately. No subsequent line, including the YB computation, risk update, context decision, or Accept return, is executed. Therefore the YB-IoT-CG layer creates no new control-flow path from failed base authentication to acceptance. Any unauthenticated acceptance must arise from a false acceptance in ${\mathrm{Auth}}_{0}$ or from an implementation fault not represented by the pseudocode. □

**Proposition** **13** (Accepted-message safety invariant)**.** *If Algorithm 1 returns Accept for message $M_{i}$, then all of the following conditions hold at the decision point:*

*1.*  *$M_{i}$ is well formed;*
*2.*  *$\kappa_{i}\notin R_{\mathrm{cache}}$;*
*3.*  *${\mathrm{Auth}}_{0}\left(M_{i}\right)=1$;*
*4.*  *$L_{i}=R_{i}^{\mathrm{YB}}$;*
*5.*  *$R_{i}<\theta_{\mathrm{risk}}$; and*
*6.*  ContextDecision *$(d_{i},R_{i},{\mathrm{ctx}}_{i})=\mathrm{Accept}$.*

**Proof.**  The result follows by backward reasoning from the unique Accept return. Reaching that line requires bypassing every earlier return. Therefore the malformed-message test is false, the revocation-cache test is false, base authentication succeeds, and the YB mismatch test is false. It also requires the $D_{i}=\mathrm{Revoke}$ branch to be false and the challenge branch $\left(R_{i}\geq \theta_{\mathrm{risk}}\right)\vee \left(D_{i}=\mathrm{Challenge}\right)$ to be false. Since Algorithm 3 returns only Accept, Challenge, or Revoke, the remaining context value is Accept, and the negated risk inequality gives $R_{i}<\theta_{\mathrm{risk}}$. □

**Proposition** **14** (Unauthenticated traffic cannot enqueue a new revocation)**.** *Assume the revocation queue is modified only by the explicit *QueueRevocation* operation in Algorithm 1. Then a message that fails ${\mathrm{Auth}}_{0}$ cannot enqueue a revocation for any DID.*

**Proof.**  The sole QueueRevocation call occurs after successful VerifyBaseAuth, successful YB consistency, computation of the bounded risk score, a context result $D_{i}=\mathrm{Revoke}$, and $R_{i}\geq \theta_{\mathrm{revoke}}$. A base-authentication failure returns before all of these lines. The early cache branch may *recognize* an already-published revocation, but it does not create or enqueue a new revocation transaction. □

Let $n_{i}=\left|C_{i}\right|$ denote the number of bytes processed by the canonical digest after any already-available payload digest is incorporated, and let *K* be the maximum number of risky endpoints inspected in the selected bucket. Assume hash processing is linear in input bytes, each modular operation on the fixed machine-word representation has constant cost, revocation-cache lookup has expected O(1) cost, and the number of context predicates is a deployment constant *m*.

**Proposition** **15** (Incremental time complexity)**.** *Under the assumptions above, the incremental post-authentication decision cost satisfies*

(68) $$T_{\mathrm{YB},\mathrm{inc}}\left(n_{i},K\right)=\Theta \left(n_{i}\right)+\Theta \left(K\right)+O\left(1\right).$$

*If $n_{i}\leq n_{\max}$ and $K\leq K_{\max}$ are deployment constants, then $T_{\mathrm{YB},\mathrm{inc}}=O\left(1\right)$ with respect to traffic-history length, observation-window length, training-set size, and number of previous packets. If raw payload hashing is included and payload size is variable, the dominant digest term is $\Theta (|p_{i}|)$.*

**Proof.**  The canonical digest scans $n_{i}$ bytes, giving $\Theta (n_{i})$. The reduction performs exactly three fixed-word modulo operations. Each YB path uses exactly three evaluations of $r_{s}$, hence six pairwise evaluations total, all constant-size. The revocation cache lookup is expected O(1). The proximity score computes at most *K* three-coordinate toroidal distances, giving Θ(K) when the bucket is nonempty. Freshness, a fixed number *m* of context predicates, convex aggregation, and threshold comparisons contribute O(1) for fixed policy size. Summing the terms yields Equation (68). Fixing $n_{i}$ and *K* removes dependence on any growing history variable. □

The simulation’s 40-operation value is therefore a profile-specific operation proxy, not a universal instruction count and not an energy measurement. The asymptotic proposition and the measured/proxy quantities answer different questions.

### 7.1. Memory and Audit-Proof Complexity

For a fixed revocation cache implementation, per-message temporary algebraic memory is O(1) because two triples and a constant number of scores are retained. A risk bucket requires O(K) entries, bounded by policy. A Merkle inclusion proof for a batch of *B* leaves contains $\left\lceil \log_{2}B\right\rceil$ sibling hashes for a balanced binary tree and is therefore O(logB) in proof size and verification hashes. The off-chain archive itself grows with the number of decisions; the protocol does not claim constant total audit-storage growth.

### 7.2. Formal Claim Matrix

Table 4 summarizes what is proved and what remains assumption-dependent.

### 7.3. Scope of Formal Assurance

The strengthened analysis proves the properties that follow from the specified algebra, control flow, bounded policy functions, Merkle construction, and conditional ledger model. A full end-to-end adversarial game for forgery, key compromise, enrollment, remote attestation, malicious context sources, Byzantine gateway coalitions, concrete Fabric endorsement policies, and recovery semantics would require fixing those mechanisms at implementation level. Such a proof is therefore not fabricated here. Instead, the manuscript provides explicit assumptions, theorem-level guarantees for the instantiated mathematical core, and a claim matrix that identifies the remaining trust boundaries.

### 7.4. Resistance to Forged Credential Transcripts

Let $\left(a_{i},b_{i},c_{i}\right)\in Z_{q}^{3}$ be the reduced credential transcript obtained from the digest of an IoT message. The gateway computes two traversal outputs:

(69) $$\begin{array}{cc} L_{i} & =\left(r⊗I\right)\left(I⊗r\right)\left(r⊗I\right)\left(a_{i},b_{i},c_{i}\right), \end{array}$$

(70) $$\begin{array}{cc} R_{i}^{\mathrm{YB}} & =\left(I⊗r\right)\left(r⊗I\right)\left(I⊗r\right)\left(a_{i},b_{i},c_{i}\right). \end{array}$$

A credential transcript passes the algebraic comparator only if $L_{i}=R_{i}^{\mathrm{YB}}$. By Proposition 6, this equality holds for every correctly recomputed external input, including an externally modified one. The positive rejection statement is therefore restricted to the isolated one-path perturbation model of Proposition 7; when that perturbation causes the outputs to diverge, the gateway challenges the message and can store both final states as explainable audit evidence. External-message forgery resistance remains a property of ${\mathrm{Auth}}_{0}$, not of the YB equality.

### 7.5. Replay and Freshness Control

Replay resistance is supported by the inclusion of the nonce $n_{i}$, timestamp $t_{i}$, firmware evidence ${\mathrm{fw}}_{i}$, and contextual metadata ${\mathrm{ctx}}_{i}$ in the credential digest. A replayed message may preserve a syntactically valid token, but it changes the freshness profile evaluated by $R_{\mathrm{fresh}}\left(n_{i},t_{i}\right)$. Consequently, replayed or stale credentials can be challenged or revoked even when the algebraic transcript remains internally consistent.

### 7.6. Credential Cloning and Contextual Inconsistency

A cloned IoT credential may reuse a valid device identifier or token from a legitimate node. However, the proposed risk model also includes contextual information through $R_{\mathrm{ctx}}\left(d_{i}\right)$, which can represent gateway identifier, location class, firmware version, device cluster, maintenance state, or operational time window. If a cloned credential is used from an inconsistent context, the gateway can increase the risk score and return a challenge or revocation decision according to the active Zero-Trust policy. Because the identifier is a ledger-anchored DID, a clone revoked at one gateway is rejected at every other consortium gateway within the staleness bound of Proposition 11.

### 7.7. Ledger-Backed Non-Repudiation and Revocation Integrity

A single compromised gateway may attempt to suppress, rewrite, or replay security verdicts. Under the assumptions of Section 3, three properties follow from the blockchain layer. First, *tamper evidence*: any modification of stored evidence $E_{i}$ is detectable, because its Merkle inclusion proof no longer verifies against the anchored root $B_{j}$ [5,33]. Second, *non-repudiation*: anchor and revocation transactions are signed by the issuing gateway and totally ordered by the ledger, so a gateway cannot later deny having issued a verdict [6]. Third, *fork-free revocation*: the monotone Revoked status and the quorum-gated reinstate transition prevent a minority coalition from reverting a revocation, so all honest gateways converge to a single global revocation view. These properties hold as long as the adversary does not control an endorsement quorum, which is the standard trust assumption of permissioned ledgers.

### 7.8. Bounded Kernel Complexity

The Yang–Baxter verification step is applied to a fixed-size transcript $\left(a_{i},b_{i},c_{i}\right)$. Since the number of pairwise transformations is constant, the kernel cost does not grow with the number of previous packets, traffic-window length, feature-vector dimension, or training-set size. The per-packet verification complexity of the algebraic kernel is therefore O(1), excluding standard digest computation and optional contextual policy checks. The revocation-cache lookup is an O(1) expected-time set-membership test, and all ledger interaction is asynchronous, so blockchain latency does not enter the packet decision path. This bounded cost is central for edge gateways that must authenticate large streams of IoT messages under latency and memory constraints.

### 7.9. Auditability and Explainability

For each evaluated message, the gateway can store compact audit evidence $E_{i}=\left(h_{i},R_{i},{\mathrm{Decision}}_{i}\right)$, together with the traversal outputs when a mismatch occurs. Since the rejection cause can be represented as a concrete algebraic inconsistency or as an explicit risk-threshold violation, the decision is more explainable than purely black-box anomaly scores. The evidence is signed, summarized in a Merkle tree, and anchored on the permissioned blockchain, enabling third-party verification with logarithmic-size inclusion proofs.

The Merkle guarantee is limited to submitted evidence (Proposition 9); it does not prove completeness for events that a compromised gateway suppresses before batching.

### 7.10. Complementarity with Standard Authentication

YB-IoT-CG is designed as a complementary security layer. Standard mechanisms such as MACs, digital signatures, certificates, TLS, DTLS, MQTT security, secure elements, or device attestation remain necessary for cryptographic identity verification. The proposed kernel adds deterministic transcript consistency, risk scoring, ledger-verifiable revocation, and explainable rejection before cloud ingestion.

## 8. Experimental Setup

This section presents the experimental methodology used to evaluate the proposed Yang–Baxter IoT Consistency Gateway (YB-IoT-CG) through a controlled Python-based simulation. The evaluation does not correspond to a physical IoT deployment or to a full reproduction of a conventional intrusion-detection benchmark. Instead, it simulates representative packet-level authentication scenarios in which legitimate and adversarial IoT messages are generated under controlled traffic, delay, and attack conditions, following a simulation-oriented evaluation logic [43]. The blockchain layer is simulated through a ledger model parameterized with commit-latency and event-propagation distributions representative of permissioned platforms [6].

This section evaluates the *incremental post-authentication* YB-IoT-CG layer through a controlled Python simulation. It is not a physical IoT deployment, a hardware-energy benchmark, a large-scale DoS experiment, a live Hyperledger Fabric test, or a source-code reproduction of the compared publications. The simulator generates representative packet-level conditions after a common lower authentication precondition and evaluates the additional algebraic/risk decision path under controlled traffic, delay, and attack classes [43]. The ledger is represented by a parameterized model of commit and event-propagation delays [6]. Consequently, all absolute latency, operation-count, accuracy, and ledger results in this section are simulation outputs under the declared model and should not be interpreted as measurements of the cited systems on their original hardware.

### 8.1. Research Questions and Hypotheses

The evaluation is organized around four research questions:

- **RQ1 (Delivery):** Does the deterministic Yang–Baxter kernel preserve legitimate telemetry delivery (goodput) at least as well as learning-based and delay-trust baselines under MitM pressure?
- **RQ2 (Latency and cost):** Does the kernel achieve lower and more stable decision delay and per-packet complexity than the baselines?
- **RQ3 (Effectiveness):** Does the simulator show high decision accuracy and low false rejection for the declared credential-manipulation/replay/context attack classes?
- **RQ4 (Blockchain overhead):** Is the modeled ledger service-time equivalent, logical payload amortization, and revocation-propagation delay compatible with keeping blockchain activity off the edge decision path?

The corresponding working hypotheses are that YB-IoT-CG attains non-inferior goodput and high modeled decision accuracy (RQ1, RQ3), lower incremental delay and complexity medians within the parameterized simulator (RQ2), and low amortized logical ledger cost without adding a synchronous packet-path transaction (RQ4).

### 8.2. Design Goals Under Test

The objective of the Python simulation is to verify whether the proposed deterministic algebraic credential kernel, together with the ledger layer, satisfies the main design goals of this work: (i) preservation of useful telemetry delivery, (ii) low decision delay at the edge, (iii) bounded computational complexity, (iv) stable handling of replay/context anomalies and intentionally corrupted intermediate execution states, and (v) amortized, non-blocking blockchain anchoring, following standard performance-evaluation criteria for computer systems [44].

The Python simulation generates synthetic IoT packet streams containing both legitimate and adversarial messages under controlled traffic and attack conditions. The same simulated packet-level conditions are applied to all evaluated approaches in order to enable a consistent comparison, following a controlled experimental-design rationale [45]. The evaluation compares YB-IoT-CG with two recent MitM-oriented IoT security approaches. The first one is the learning-based MitM detection framework proposed by Ali and Al-Sharafi [17], which reports machine-learning and deep-learning classifiers for MitM attack mitigation in IoT networks. The second one is the instantaneous communication trust framework proposed by Basri et al. [20], which evaluates MitM behavior through communication-delay estimation and trust modeling. These works were selected because they represent two dominant families of MitM detection in IoT: learning-based traffic classification and delay-trust communication analysis.

Unlike these baselines, YB-IoT-CG does not infer attacks from traffic patterns, training data, or temporal sequence behavior. Instead, it performs deterministic credential verification using a fixed algebraic consistency relation. Therefore, the Python-based comparison focuses on metrics that capture operational behavior at the edge under simulated IoT conditions: goodput, decision delay, kernel complexity, detection accuracy, false rejection rate, the goodput–complexity trade-off, and the blockchain-anchoring overhead.

### 8.3. Statistical Summary Protocol

Each configuration is repeated over 60 independent runs with distinct random seeds. Randomness is controlled through a single master seed (20260705) expanded into independent substreams for the shared packet stream, profile-specific simulator noise, and the ledger model. For every metric, the median, interquartile range, and bootstrap 95% confidence interval of the median (10,000 resamples) are reported. Because Ali-RF and Basri-IPCTCM are synthetic methodological profiles rather than reproduced implementations measured at the same system boundary, no cross-method hypothesis test is used to support superiority, non-inferiority, or speedup claims. Inferential comparisons across those profiles were removed from the evidentiary argument; the remaining cross-profile values are descriptive outputs of the configured simulator scenario only [43,44,45].

### 8.4. Comparative Baselines

Three approaches are compared in the evaluation. The first baseline corresponds to the best reported learning-based MitM detection profile from Ali and Al-Sharafi [17]. Since the original trained models, datasets, feature vectors, and hyperparameters are not fully reproduced in this work, this baseline is implemented as a reported classifier-level profile. In the simulation, this profile represents the behavior of a Random-Forest-based MitM detector with high detection accuracy but with non-negligible inference and feature-processing cost.

The second baseline corresponds to the IPCTCM delay-trust model proposed by Basri et al. [20]. Rather than repeating the mathematical formulation already presented in that work, this paper uses it as a delay-trust communication baseline. In the simulation, the baseline represents the reported methodological behavior of IPCTCM: expected communication-delay estimation, instantaneous deviation analysis, trust scoring, and threshold-based MitM decision.

The third approach is the proposed Baxter/YB-IoT-CG deterministic credential kernel with blockchain anchoring. This kernel evaluates whether a packet credential transcript satisfies a fixed algebraic consistency condition. Given the same credential transcript, the same decision is always produced. This deterministic behavior is the main reason why the proposed kernel can reduce decision delay, computational complexity, and decision variability, while the asynchronous ledger client keeps anchoring off the packet path.

The cited papers define methodological families rather than reproduced implementations. The numerical values used by the Ali-RF and Basri-IPCTCM profiles below—including their simulator accuracies, latency distributions, and operation-count proxies—are outputs of the common synthetic simulator and are not the published benchmark values of the source papers. Accordingly, cross-profile ratios and statistical tests are treated only as descriptive properties of this configured simulator scenario; they are not evidence of method-level superiority over the cited external works. Table 5 summarizes the comparative evaluation framework.

### 8.5. Experimental Factors, Levels, and Parameters

The experiment was designed as a controlled packet-level simulation. Each run generates a stream of IoT packets containing legitimate and adversarial messages. The same synthetic packet stream is supplied to all profiles to control scenario input; this does not make the methods methodologically equivalent because their implementations and measurement boundaries differ.

The simulated IoT message is represented as

(71) $$M_{i}=\left(d_{i},\tau_{i},n_{i},t_{i},p_{i},\sigma_{i}\right),$$

where $d_{i}$ is the device identifier, $\tau_{i}$ is the session token, $n_{i}$ is the nonce, $t_{i}$ is the credential/message timestamp, $p_{i}$ is the useful payload, and $\sigma_{i}$ is the MAC or digital signature generated by the lower authentication layer. Packet arrival time is represented separately by $t_{\mathrm{arrival},i}$. The simulator evaluates the incremental YB-IoT-CG layer after successful base authentication; it does not reinterpret $\tau_{i}$ or $\sigma_{i}$.

The experimental factors and their levels are summarized in Table 6. These factors were selected to capture the conditions that most directly affect credential verification, MitM detection, and ledger interaction: traffic volume, attack intensity, useful payload size, observation window, decision-kernel type, anchoring batch size, and ledger commit latency, which are treated as controlled experimental factors [45].

**Risk-policy parameter disclosure for reproducibility.** The following values are the exact risk-policy parameters used in the reported simulation and have been cross-checked against the original simulation notebook. They are published here to make the configured policy and its update behavior fully reproducible; the reported figures and summary statistics therefore remain unchanged. Table 7 lists these parameters.

### 8.6. Rationale for Metric Selection

The selected metrics were chosen because they directly evaluate the design claims of YB-IoT-CG and follow standard system-performance evaluation practice [44].

First, goodput measures the amount of legitimate telemetry that is accepted and delivered to the application layer. This is essential because an edge security mechanism should not only reject malicious traffic, but also preserve useful data delivery.

Second, decision delay measures the time introduced by the security kernel before a packet is accepted or rejected. This metric is critical in IoT environments, where edge devices frequently operate under strict latency constraints.

Third, kernel complexity measures the computational effort required per packet. This metric is necessary because the proposed mechanism targets resource-constrained environments, where recurrent inference, long traffic windows, or high-dimensional feature extraction may be expensive.

Fourth, detection accuracy is included to preserve comparability with classification-oriented MitM detection studies. Although YB-IoT-CG is not a conventional classifier, accuracy provides a general measure of correct acceptance and rejection decisions.

Fifth, false rejection rate is included because excessive rejection of legitimate packets directly reduces useful telemetry delivery and can damage IoT service continuity.

Sixth, three blockchain-oriented metrics quantify the ledger layer: amortized ledger service-time equivalent per decision ($t_{\mathrm{tx}}/B$), logical on-chain payload per decision, and empirically modeled revocation propagation time (interpreted under Proposition 11), together with audit completeness, defined as the fraction of decisions covered by a verifiable Merkle inclusion proof against an anchored root.

Finally, the goodput–complexity trade-off summarizes the main contribution of the paper: YB-IoT-CG seeks to preserve useful traffic while maintaining a small and deterministic decision kernel whose verdicts are ledger-verifiable.

### 8.7. Goodput Metric

Goodput measures only useful accepted traffic. Unlike throughput, which counts all transmitted traffic, goodput excludes rejected packets and malicious packets. For the evaluated IoT security mechanisms, goodput is defined as

(72) $$G=\frac{\sum_{i=1}^{N}p_{i}\cdot I\left(D_{i}=\mathrm{Accept}\right)\cdot I\left(M_{i}=\mathrm{Legitimate}\right)}{\Delta T},$$

where $p_{i}$ is the useful payload size, $D_{i}$ is the security decision, I(·) is the indicator function, $M_{i}$ is the true packet condition, and ΔT is the observation interval.

Higher goodput indicates that a larger amount of valid telemetry reaches the application layer. In this evaluation, goodput is especially important because YB-IoT-CG is deterministic: valid authenticated credential transcripts are consistently accepted, while modeled replay/context anomalies and intentionally inconsistent intermediate states are challenged or rejected before cloud admission.

### 8.8. Decision Delay

Decision delay measures the latency introduced by the security mechanism. For message $M_{i}$, it is defined as

(73) $$\Delta t_{i}=t_{\mathrm{decision},i}-t_{\mathrm{arrival},i}.$$

For the proposed framework, the delay can be decomposed as

(74) $$\Delta t_{i}^{\mathrm{YB}}=t_{H}+t_{\rho }+t_{\mathrm{YBE}}+t_{D},$$

where $t_{H}$ is digest computation time, $t_{\rho }$ is algebraic reduction time, $t_{\mathrm{YBE}}$ is Yang–Baxter consistency verification time, and $t_{D}$ is decision-policy execution time. Ledger anchoring is asynchronous and therefore does not appear in Equation (74); its amortized cost is reported separately through Equation (65).

The reported $\Delta t_{i}^{\mathrm{YB}}$ is the incremental post-authentication stage. A complete deployment additionally incurs the pre-existing $t_{{\mathrm{Auth}}_{0}}$ cost. Consequently, the 7.8 μs result is not an end-to-end credential-authentication time, and $t_{\mathrm{tx}}/B$ is not individual audit-verifiability latency.

For the learning-based baseline, the delay is described in terms of feature extraction and model inference, following the methodological profile reported by Ali and Al-Sharafi [17]. For the delay-trust baseline, the delay is associated with communication-delay estimation and trust-decision computation, as reported by Basri et al. [20]. This avoids restating the full internal formulations of the compared works and keeps the analysis focused on the proposed kernel.

### 8.9. Computational Complexity Analysis

The third metric evaluates the computational complexity of each decision kernel.

For the learning-based baseline, the decision cost is interpreted according to the classifier-based detection pipeline reported by Ali and Al-Sharafi [17]. In general terms, this cost is associated with traffic-feature preparation and model inference. Since the original trained model, dataset, and implementation parameters are not reproduced here, the baseline is used as a reported learning-based profile rather than as a source-code reproduction.

For the Basri et al. delay-trust baseline, the cost is interpreted according to the IPCTCM communication-trust procedure [20]. Instead of repeating the mathematical equations already provided in that article, the present work represents the baseline at the kernel-profile level, considering delay estimation, trust-score computation, and decision thresholding as the main sources of computational cost.

For the proposed YB-IoT-CG kernel, the credential digest is reduced to a fixed algebraic transcript

(75) $$\rho_{q}\left(h_{i}\right)=\left(a_{i},b_{i},c_{i}\right).$$

The deterministic Yang–Baxter consistency condition is then evaluated over this fixed transcript. Since the transcript size is fixed, the number of pairwise algebraic operations does not grow with traffic history, sequence length, or model size. Therefore, the Yang–Baxter verification stage has bounded kernel cost with respect to the number of packets.

The total proposed kernel cost can be summarized as the combination of digest processing, algebraic reduction, consistency checking, revocation-cache lookup, and decision execution. This bounded-complexity behavior, combined with the O(1/B) on-chain storage of Equation (65), is one of the main expected advantages of the proposed framework.

Proposition 15 refines this statement to $\Theta (|C_{i}|)+\Theta \left(K\right)+O\left(1\right)$. Fixed record size and deployment-bounded *K* yield O(1) only with respect to growing history/window/training-set variables; variable raw-payload hashing is $\Theta (|p_{i}|)$. The 40-operation value used by the simulator is a profile-specific proxy and is not a battery-energy measurement.

### 8.10. Reproducibility

All simulation code, the seed-derivation tree, kernel-profile parameters, ledger-model parameters, and figure-generation scripts are packaged as a single Python notebook that regenerates every reported figure, table, and statistical test deterministically from the master seed with one command (“Run all”). The notebook exports the raw per-run results, the seed manifest, the recorded software environment, and the summary statistics as machine-readable files; all deterministic outputs are bit-identical across executions, and the only non-deterministic quantity is the wall-clock timing of the measured YB kernel, which remains sub-millisecond on commodity hardware. The artifact is available from the corresponding author upon reasonable request and is intended for public release upon acceptance.

## 9. Results and Discussion

### 9.1. Simulation-Based Validation and Figures

The evaluation generates six figures that directly support the claims of the article. These figures are selected because they jointly evaluate useful traffic delivery, latency, computational cost, security effectiveness, preservation of legitimate packets, and the trade-off between useful delivery and complexity. Table 8 reports descriptive summaries of the five packet-level metrics over the 60 seeded runs. Cross-profile hypothesis-test columns have been removed because the external methods are not reproduced at an equivalent measurement boundary. The blockchain-anchoring overhead is reported separately in Section 9.2.

The labels Ali-RF and Basri-IPCTCM in the result figures/tables identify simulator profiles inspired by the corresponding methodological families. Their numerical values (including 96.50% and 98.10% simulated accuracy, the delay distributions, and operation-count proxies) are *not* presented as the published values of the cited papers.

**Figure 3:**
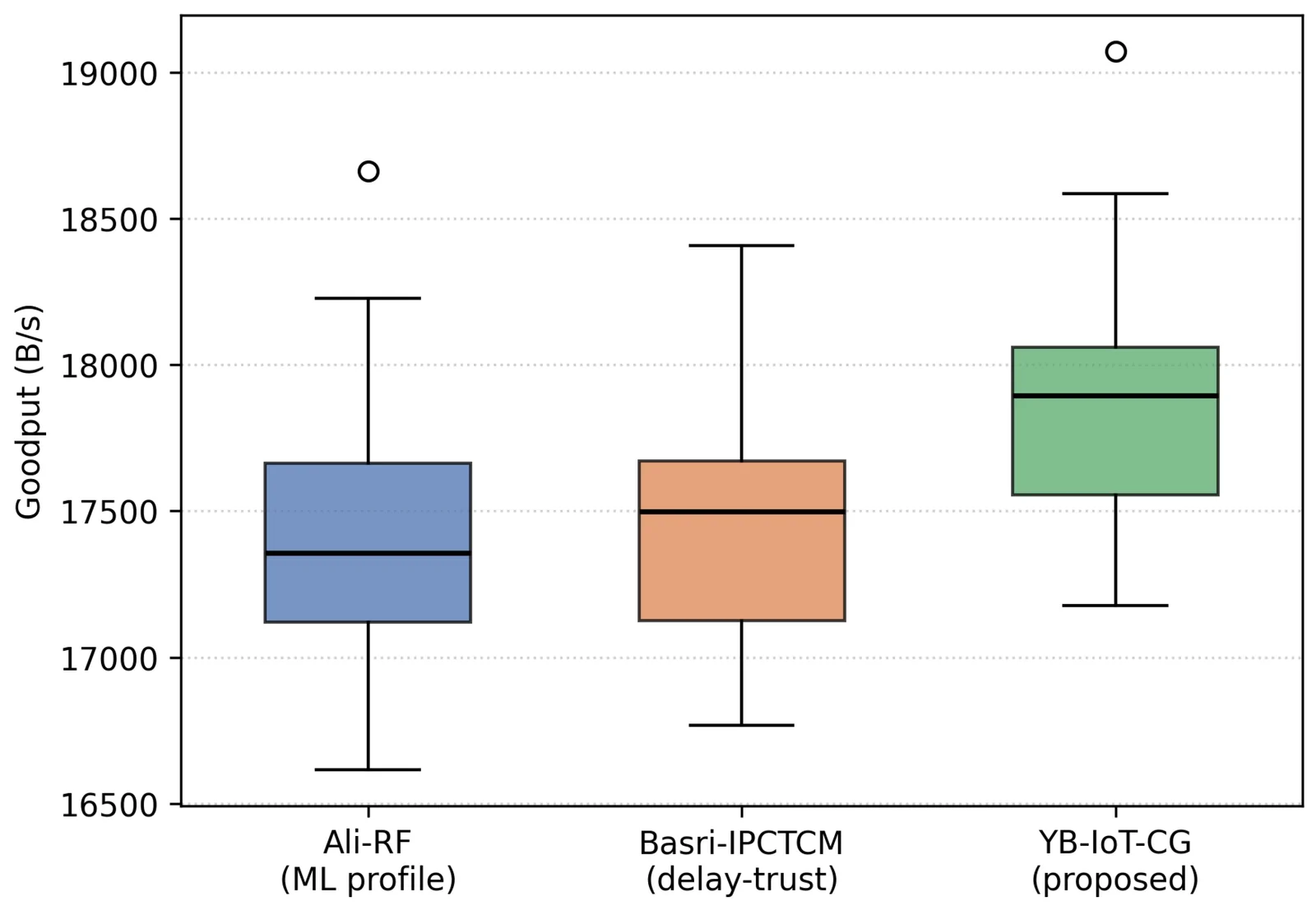
Goodput comparison among the Ali-RF simulator learning-based profile, the Basri-IPCTCM simulator delay-trust profile, and the proposed YB-IoT-CG deterministic credential kernel.

Figure 3 reports the goodput generated by the three simulator profiles under the common synthetic traffic scenario. The corresponding medians are 17,894.4 B/s for YB-IoT-CG, 17,356.8 B/s for Ali-RF, and 17,497.6 B/s for Basri-IPCTCM. These differences are reported descriptively only. Because Ali-RF and Basri-IPCTCM are parameterized profiles rather than reproduced implementations, neither the percentage gaps nor the associated rank tests are interpreted as evidence that YB-IoT-CG is superior to the cited methods.

**Figure 4:**
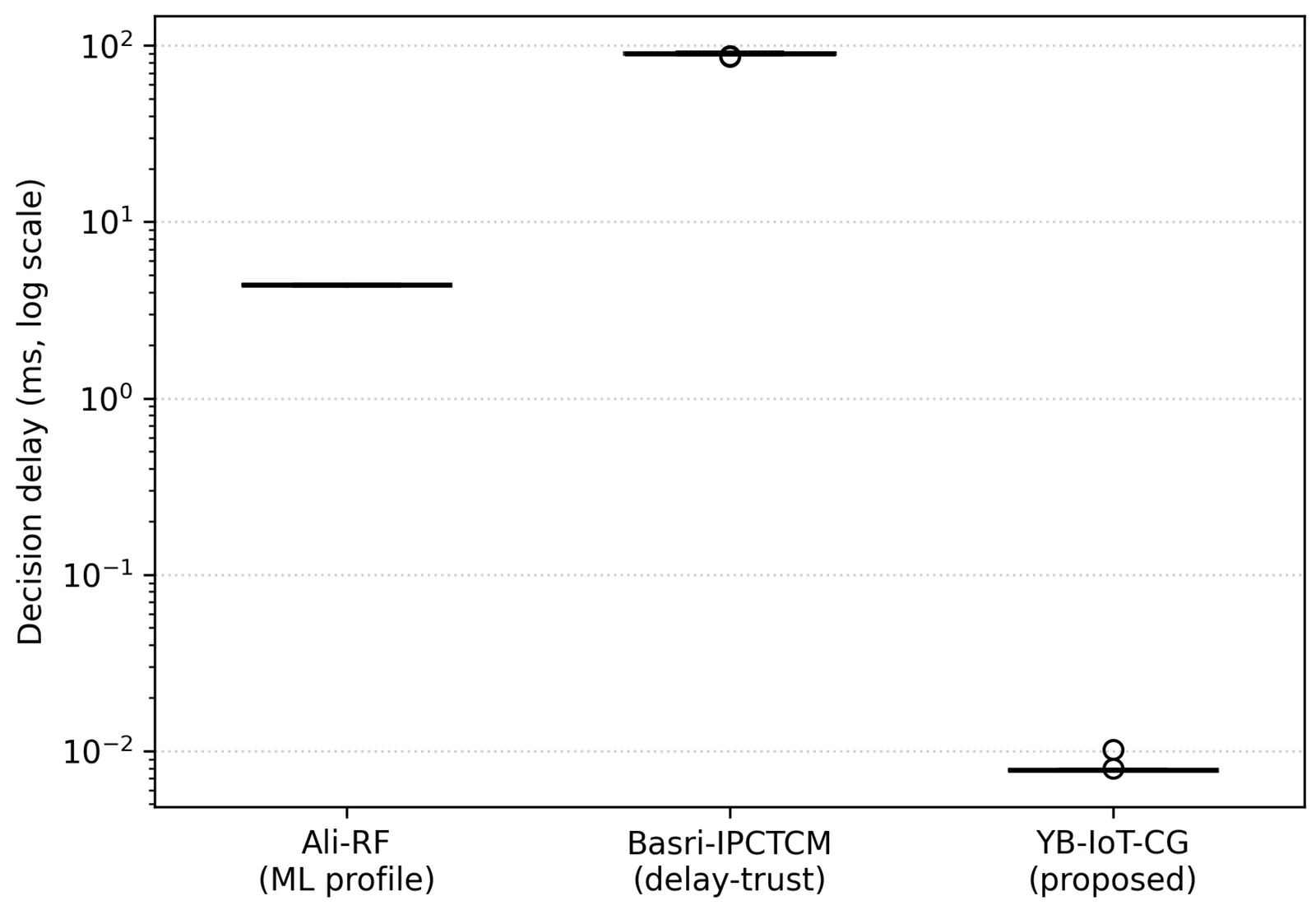
Decision-delay comparison among the evaluated kernels.

Figure 4 reports simulator-profile decision delay on a logarithmic scale. The YB-IoT-CG value is the measured wall-clock time of the complete incremental post-authentication kernel (digest, reduction, double traversal, and policy), with a median of 7.8 μs; it is not end-to-end authentication latency and not YB-only traversal latency. The Ali-RF and Basri-IPCTCM values arise from different synthetic profile definitions and measurement semantics. They are therefore shown for internal scenario characterization only; speedup factors and cross-method significance claims are not used to establish superiority over the external works.

**Figure 5:**
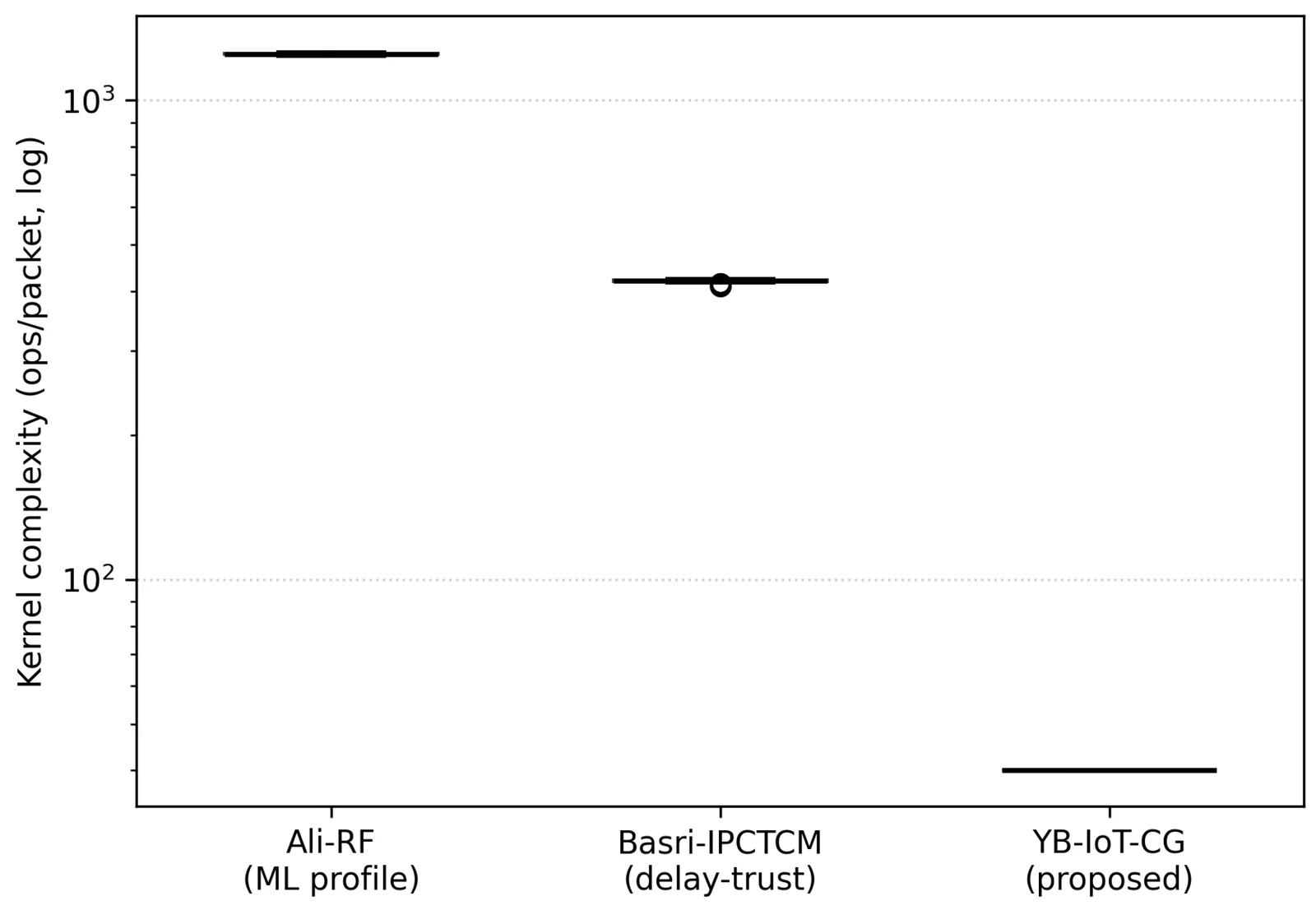
Kernel-complexity comparison in operations per packet.

Figure 5 reports the operation-count proxies used by the simulator. The YB-IoT-CG profile uses a fixed 40-operation proxy for the configured record size and endpoint bound. This supports only the internal bounded-cost property formalized in Proposition 15; the 420.2 and 1251.0 operation values belong to synthetic baseline profiles and are not reproduced hardware or source-code measurements. Ratios between these proxies are therefore descriptive scenario quantities, not method-level complexity speedups.

**Figure 6:**
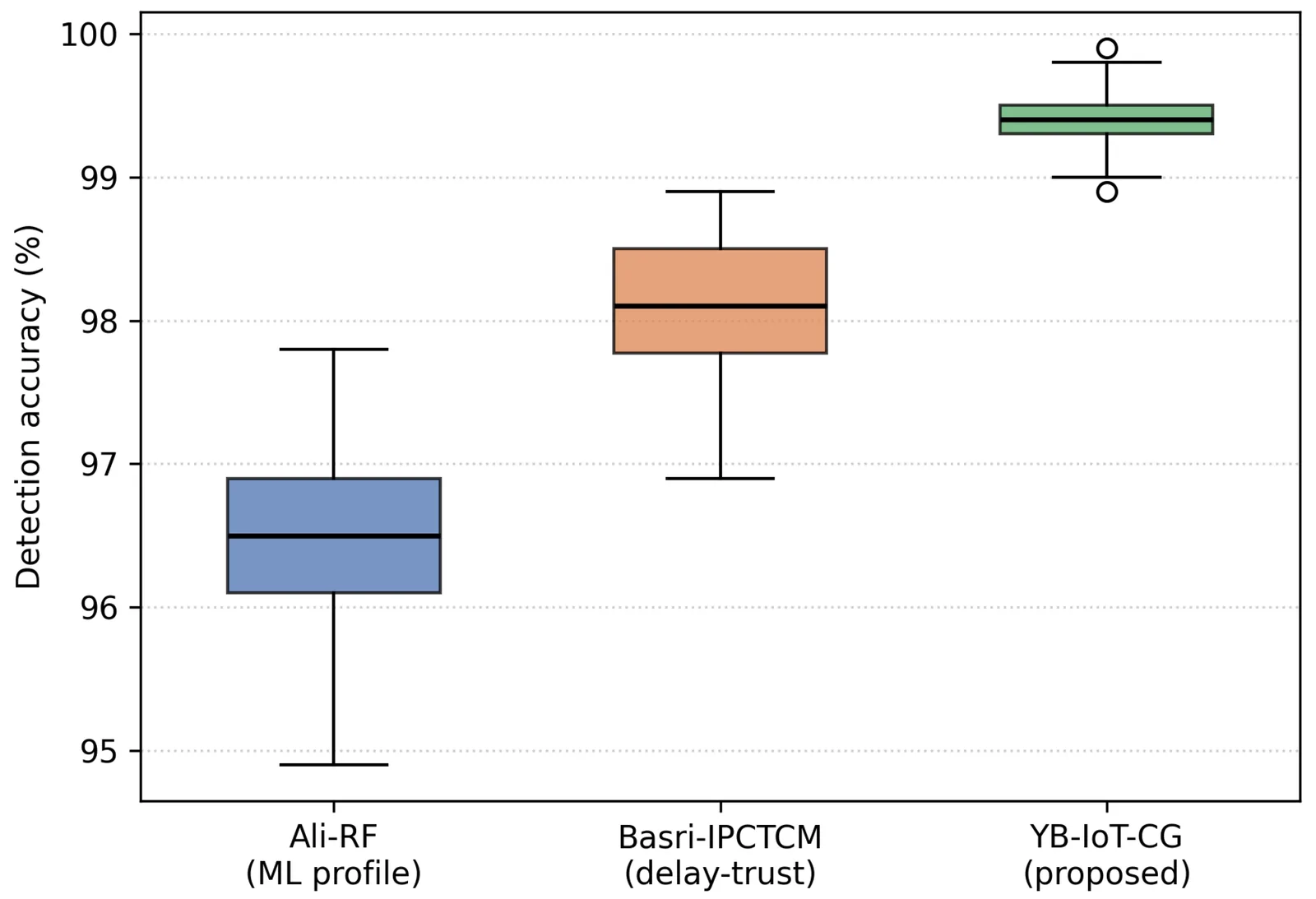
Detection-accuracy comparison among the evaluated approaches.

Figure 6 reports aggregate decision accuracy for the configured simulator profiles. YB-IoT-CG reaches a median of 99.40% for the complete composed policy. This value must not be attributed to the Yang–Baxter stage alone: replay detection is produced by freshness/nonce state, clone detection by contextual policy, and only the explicitly injected isolated intermediate-state corruption is assigned to YB path divergence. The Ali-RF and Basri-IPCTCM percentages are simulator-profile outputs rather than replicated published measurements; consequently, cross-profile significance tests are not interpreted as evidence of external-method superiority.

**Figure 7:**
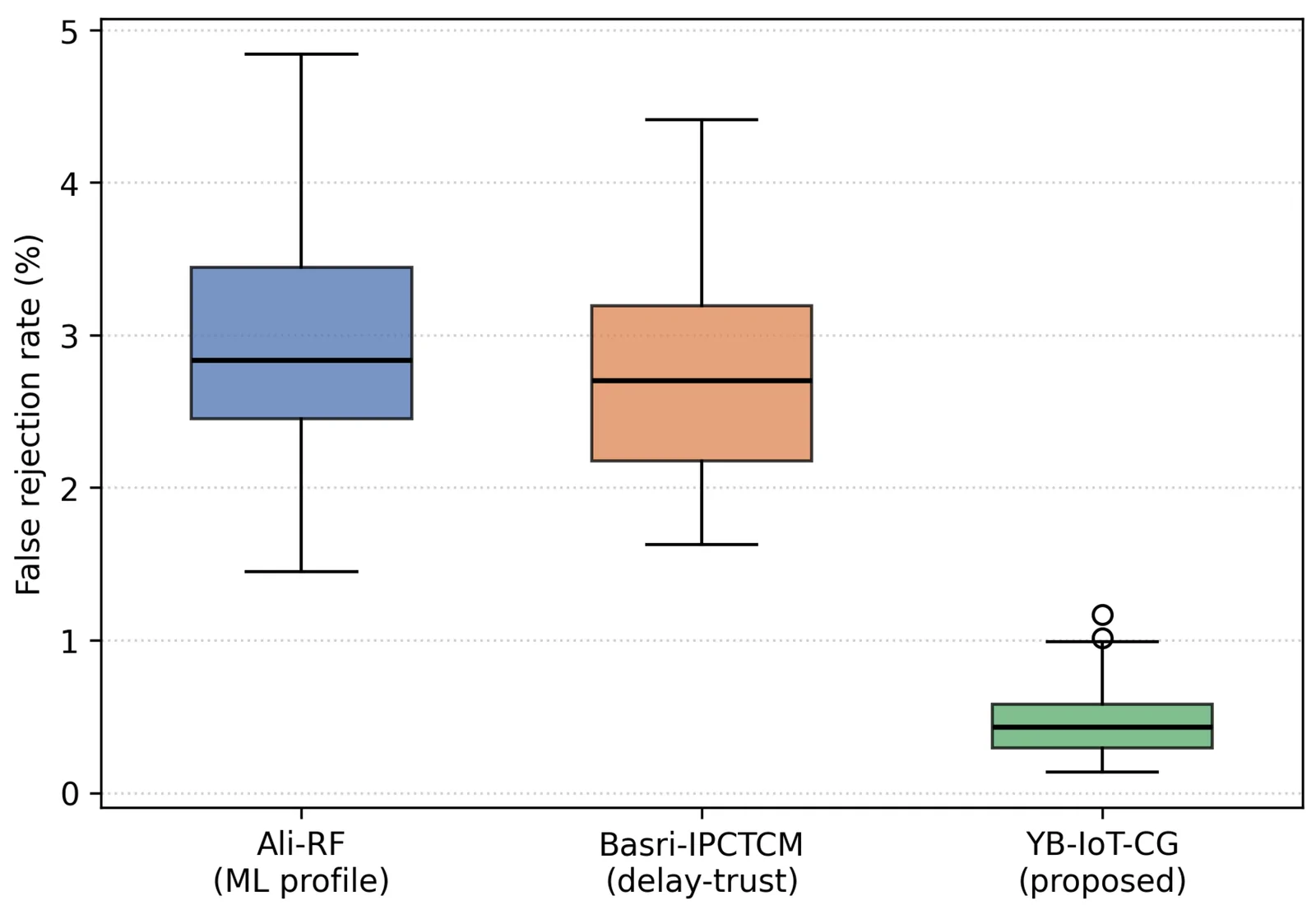
False rejection rate for legitimate packets.

Figure 7 reports false rejection within the configured simulator. The YB-IoT-CG composed policy has a median false rejection rate of 0.43%, while the two synthetic comparison profiles produce 2.83% and 2.70%. These values are retained as internal scenario outputs only; no cross-method superiority claim is made. Residual YB-IoT-CG false rejections correspond to legitimate maintenance traffic whose contextual anomaly produces a Challenge, which can be recovered through re-authentication.

**Figure 8:**
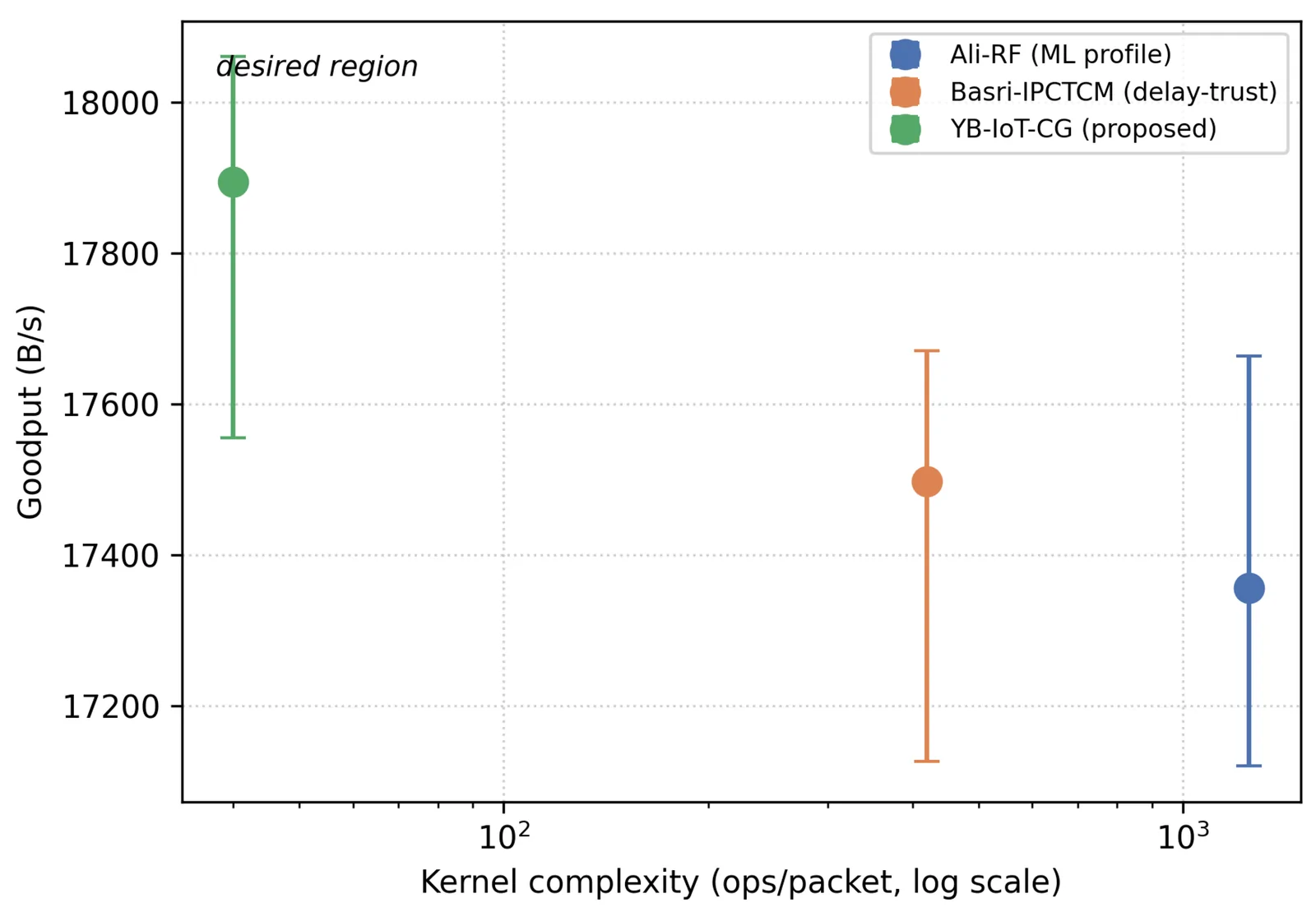
Goodput–complexity trade-off among the evaluated kernels.

Figure 8 visualizes the internal trade-off produced by the configured simulator profiles. It is descriptive rather than a methodologically equivalent benchmark: the baseline coordinates are synthetic profile outputs, whereas YB-IoT-CG is measured as the local post-authentication kernel. The figure is therefore used to illustrate the scenario design space and not to claim general superiority, speedup, or statistically established dominance over the cited external methods.

### 9.2. Blockchain Anchoring and Revocation Overhead

Table 9 summarizes the *modeled logical ledger* results under the parameters of Table 6. For B=64 and a mean modeled commit time of 2.0 s, $t_{\mathrm{tx}}/B$ has a median of 31.4 ms [26.7–35.5]. Following the revised terminology of Section 6.6, this value is an amortized ledger service-time equivalent; it is not the time at which an individual decision becomes verifiable on-chain. Individual verifiability also includes batch-fill/flush waiting and commit time. Likewise, 0.81 B/decision is only the logical application payload (32-byte root plus 20 bytes of modeled metadata amortized over 64 decisions); transaction envelopes, signatures, indexing, block headers, endorsements, and replication are not included.

Exactly ⌈1000/64⌉=16 anchor transactions are emitted per run after the closing batch is flushed. Modeled revocation propagation has a median of 2.15 s [1.88–2.42], which is reported as an empirical distribution rather than as a deterministic bound because the simulated commit and event delays are stochastic. Audit completeness reaches 100% in the simulator because Algorithm 1 emits evidence for all three decision branches and the final partial batch is flushed. The packet path performs no synchronous ledger transaction.

### 9.3. Analysis of Results

Within the declared parameterized simulator, the results support the narrower hypothesis that a deterministic post-authentication credential-screening layer can provide a low-complexity complement to traffic-centric MitM detection while keeping the modeled ledger path asynchronous. Each research question of Section 8.1 can now be answered against its working hypothesis.

**RQ1 (Delivery).** Within the configured common simulator, the YB-IoT-CG profile produces a median goodput of 17,894.4 B/s versus 17,356.8 and 17,497.6 B/s for the two synthetic comparison profiles. This is an internal scenario result only; it does not establish non-inferiority or superiority with respect to the cited external implementations.

**RQ2 (Latency and cost).** The complete YB-IoT-CG post-authentication kernel has a median measured decision time of 7.8 μs and a configured 40-operation proxy. Because the external profiles use different simulated semantics and are not source-code reproductions, the cross-profile ratios are not interpreted as speedup factors. The supported claim is narrower: the proposed kernel has bounded incremental cost with respect to traffic history for fixed record size and endpoint bound *K*, and ledger interaction is asynchronous.

**RQ3 (Effectiveness and mechanism attribution).** The composed YB-IoT-CG policy reaches 99.40% median aggregate decision accuracy and 0.43% median false rejection in the declared simulator. Mechanism attribution is explicit: ${\mathrm{Auth}}_{0}$ supplies authenticity, freshness detects replay/staleness, contextual policy addresses clone/context anomalies, the registry propagates revocation, and the YB comparator detects only the restricted isolated intermediate-state perturbation of Proposition 7. Proposition 6 precludes attributing arbitrary external-forgery detection to Yang–Baxter equality.

**RQ4 (Blockchain overhead).** The simulation supports the narrower claim that the ledger path can remain decoupled from packet admission. Table 9 reports a 31.4 ms amortized service-time equivalent and 0.81 B logical anchor payload per decision for B=64, while the packet path performs no synchronous ledger operation. The 2.15 s revocation value is an empirical modeled propagation time rather than a deterministic bound. Real Fabric transaction envelopes, endorsement overhead, physical replicated storage, partitions, and revocation storms are not measured here.

Finally, Figure 8 summarizes the configured simulator trade-off. The empirical contribution claimed here is limited to the measured behavior of the proposed post-authentication kernel and ledger model under the declared scenario; the figure is not used as a statistically equivalent benchmark against the cited external methods.

Together, Table 8, the six figures, and the blockchain-overhead table provide quantitative evidence that YB-IoT-CG is not intended to replace all forms of traffic-based intrusion detection. Instead, it offers a complementary credential-hygiene layer that is deterministic, bounded in complexity, low in latency, blockchain-anchored, and suitable for resource-constrained IoT gateways. Two honest caveats accompany these numbers: the baselines are reported methodological profiles rather than source-code reproductions (Section 10), and the YB decision-path timing is produced by the declared Python-based simulation environment rather than a physical IoT testbed, so absolute hardware latency and energy remain unmeasured.

A related direction is the integration of the OWL password-authenticated key exchange protocol into IoT application protocols such as CoAP [46]. This approach addresses authenticated session establishment, whereas YB-IoT-CG operates after the underlying authentication step and adds credential screening, revocation, and audit support. Evaluating their composition would require explicit interface and security assumptions and a common implementation; such integration was not evaluated in the present study.

## 10. Limitations

Although the proposed YB-IoT-CG framework provides a deterministic and explainable credential-screening mechanism with blockchain-anchored auditability, several limitations must be acknowledged. First, the current evaluation is based on a controlled Python simulation rather than on a large-scale physical IoT testbed. Therefore, the reported results should be interpreted as simulation-based evidence of the kernel behavior and not as final deployment measurements.

Second, the compared baselines are represented as reported methodological profiles rather than full source-code reproductions of the original works. This choice enables a controlled conceptual comparison under the same simulated packet-level conditions, but future work should implement the baselines under the same software and hardware conditions to provide a fully reproducible benchmark.

Third, the modular Yang–Baxter map used in the illustrative example is pedagogical. A production-grade deployment should evaluate alternative non-degenerate set-theoretic Yang–Baxter solutions and formally verify their suitability for credential screening, collision monitoring, and bounded edge execution.

Fourth, the risk score depends on threshold calibration and on the quality of the available contextual information. Incorrect thresholds or incomplete contextual metadata may increase false challenges or false rejections. For this reason, threshold recalibration and policy governance must be validated for each application domain.

Fifth, the blockchain layer is evaluated through a parameterized ledger model rather than a deployed Fabric or EVM network; real deployments exhibit consensus-dependent latency variability, endorsement-policy failures, ordering backpressure under bursty revocation storms, and governance costs of quorum-based reinstatement that the model only approximates. The staleness bound of Proposition 11 is conditional on explicit hard synchrony premises and also inherits the safety and liveness assumptions of the underlying ledger, and public-network realizations would add fee-market variability [42].

Finally, YB-IoT-CG does not replace conventional cryptographic authentication, network intrusion detection, firmware attestation, or physical tamper protection. It should be deployed as an additional credential-hygiene layer within a broader Zero-Trust IoT architecture.

**Additional revision-scope limitations.** The evaluation is not a physical ESP32/ARM Cortex-M testbed and does not reproduce real interrupt scheduling, radio contention, jitter, memory pressure, clock drift, secure-element latency, or battery consumption. Operation count and O(1) incremental complexity are complexity indicators, not energy measurements.

YB-IoT-CG assumes a preceding secret-based authentication layer. If that MAC/ signature/certificate/private-key layer is compromised, the Yang–Baxter stage does not restore authenticity. Proposition 6 proves that any honestly recomputed external input is YB-consistent; the positive detection guarantee is limited to Proposition 7.

The proactive risk score is deterministic and policy-based, not a trained predictor. Thresholds and risky-endpoint sets are deployment inputs, and long-horizon adaptive-threshold poisoning has not been experimentally stress-tested despite the bounded per-update rule.

The bounded-update invariant of Proposition 8 limits the displacement caused by each accepted update but does not establish resistance to cumulative adversarial drift over a long sequence of authenticated, accepted observations. No long-horizon adaptive-poisoning campaign was conducted in the present study; resistance to such poisoning is therefore not claimed and remains a limitation and topic for future work.

The Brauer construction is structural only, and $H_{B}$ is not used in any collision bound. Collision screening is expressed instead through the actual reduced-space distribution in Proposition 4; the q=997 state remains non-cryptographic, as formalized by Proposition 1.

The ledger contribution is an architectural data model and parameterized simulation, not an executed Fabric/EVM implementation. The 31.4 ms quantity is an amortized service-time equivalent and 0.81 B is logical application payload; physical transaction envelopes, endorsements, indexing, replication, partitions, missed events, and governance costs are not measured. A compromised gateway can suppress an event before submission.

The 1000-packet/30% attack setup is not a large-scale DoS, distributed-flooding, or revocation-storm benchmark. Authentication-first ordering limits unnecessary post-authentication work, but volumetric DoS resistance requires infrastructure-level controls and separate evaluation.

The external baselines are parameterized methodological profiles rather than source-code reproductions; cross-profile ratios are descriptive simulator outputs and are not presented as speedups over the cited methods.Finally, the formal propositions do not constitute a complete deployment-specific adversarial game for enrollment, key compromise, malicious context sources, Byzantine gateway coalitions, a concrete Fabric endorsement/ordering configuration, or recovery semantics.

## 11. Conclusions

This paper presented YB-IoT-CG, a deterministic and lightweight additional post-authentication security architecture for IoT environments based on Yang–Baxter algebraic consistency and a modeled permissioned-ledger design. The article was evaluated through a controlled Python-based packet-level simulation designed to analyze goodput, decision delay, kernel complexity, detection accuracy, false rejection, the goodput–complexity trade-off, and the overhead of Merkle-batched blockchain anchoring and on-chain revocation. The proposed scheme represents each device credential as a fixed algebraic transcript and verifies whether two Yang–Baxter traversal paths produce the same result. This formulation enables credential verification to be performed without traffic-history accumulation, recurrent inference, or model-dependent feature extraction, while every verdict remains verifiable against a tamper-evident ledger.

Over 60 seeded runs, the complete YB-IoT-CG post-authentication pipeline obtained a median incremental decision time of 7.8 μs, a configured 40-operation proxy for the fixed simulation profile, 99.4% aggregate decision accuracy for the declared modeled classes, and a 0.43% false rejection rate. The two external approaches are retained only as synthetic methodological profiles; their numerical outputs are not source-code reproductions and are not used to claim speedup, superiority, or statistically equivalent benchmarking. The supported complexity claim is that the proposed algebraic/policy stage has bounded cost with respect to traffic history when record size and *K* are fixed, as refined by Proposition 15. The Merkle-batched model provides 0.81 B logical anchor payload per decision and a 31.4 ms amortized ledger service-time equivalent for B=64, while the 2.15 s revocation value is an empirical modeled propagation time rather than an unconditional deterministic staleness bound.

These quantitative comparisons are outputs of the common simulator and are not measurements of ESP32/ARM hardware or source-code reproductions of the external baseline implementations. The public Yang–Baxter map does not replace the secret-bearing authentication layer; Proposition 6 gives the negative boundary explicitly, while Proposition 7 gives the scoped positive consistency result.

Overall, YB-IoT-CG should be understood as a complementary credential-hygiene layer rather than a replacement for all traffic-based intrusion-detection mechanisms. Its main contribution is a post-authentication credential-hygiene architecture that combines explicit policy enforcement, a narrowly scoped redundant execution-consistency check, and ledger-verifiable revocation/audit support. By combining algebraic consistency checking with risk-aware decision policies, contextual governance, DID-based identity binding, and Merkle-anchored auditability, the scheme provides a structured path toward more transparent and efficient IoT credential management.

Future work will validate the same architecture on ESP32- and ARM Cortex-M-class devices and industrial gateways; measure CPU, memory, battery energy, and real network jitter; integrate MQTT 5.0 over TLS 1.3 and CoAP over DTLS 1.3; deploy the registry and Merkle anchoring on a live Hyperledger Fabric consortium; evaluate batch sizes and revocation storms under real ordering backpressure; add signed-receipt/sequence or gateway-attestation mechanisms for evidence completeness; compare production-grade Yang–Baxter constructions; and develop formal composition proofs and smart-contract verification for the selected implementation.

### Research Stay and Project Context

Part of the conceptual development, architectural refinement, and international scientific collaboration associated with this work is linked to a postdoctoral research stay at the BISITE Research Group (Bioinformatics, Intelligent Systems and Educational Technology), University of Salamanca, Spain.

The research stay is conducted within the framework of the project entitled *“Arquitectura Zero-Trust y Validación Formal Inteligente para una Plataforma de NFTs con Regalías Seguras y Privacidad por Diseño en la Economía Cultural del Atlántico”*, corresponding to BPIN 2022000100083. This research project is part of the strategic initiative aimed at strengthening blockchain-based digital infrastructures and secure smart-contract technologies for the creative economy in the Department of Atlántico, Colombia.

The proposed research contributes methodological and technological knowledge in artificial intelligence, cybersecurity, privacy-preserving architectures, blockchain interoperability, trustworthy AI, and formal validation methodologies, whose scientific advances are transferable to digital health and intelligent telemedicine systems.

Although the postdoctoral research framework provides an environment for international collaboration and scientific exchange, the conception, methodology, implementation, experimental evaluation, and conclusions presented in this manuscript were independently developed by the authors and are not conditioned by the objectives of the aforementioned blockchain project.

## Figures and Tables

**Table 1:** Summarized state of the art on IoT authentication, zero trust, blockchain, edge/fog security, and privacy-preserving access control.

Year	Research Title	Proposed Solution	Main Theorems/Formal Results	Tools/Platforms	Future Work
2023	*TABI: Trust-Based ABAC Mechanism for Edge-IoT Using Blockchain Technology* [13]	Trust-based ABAC for Edge-IoT supported by blockchain.	Formal trust-score and ABAC decision model; security and performance evaluation.	Edge-IoT, blockchain, ABAC, trust model.	Improve scalability and reduce policy-evaluation overhead.
2024	*BSRA: Blockchain-Based Secure Remote Authentication Scheme for Fog-Enabled Internet of Things* [12]	Blockchain-based remote authentication for fog-enabled IoT.	Security analysis against impersonation, replay, and credential misuse.	Fog-IoT, blockchain, authentication protocol.	Optimize authentication cost and validate larger fog-IoT scenarios.
2024	*Privacy-Preserving Scheme With Bidirectional Option for Blockchain-Enhanced Logistics Internet of Things* [21]	Privacy-preserving access control for logistics IoT using blockchain and encryption.	Formal privacy/access-control design with CP-ABE and integrity verification.	Blockchain, smart contracts, CP-ABE, logistics IoT.	Improve scalability and flexible privacy-preserving queries.
2024	*A Zero-Trust Authentication Scheme With Access Control for 6G-Enabled IoT Environments* [8]	Zero-trust authentication and access control for 6G-IoT.	Security and performance analysis using blockchain and lightweight ABE.	6G-IoT, blockchain, ECC, ABE.	Reduce storage and computation costs for light IoT nodes.
2024	*Blockchain-Enhanced Zero Knowledge Proof-Based Privacy-Preserving Mutual Authentication for IoT Networks* [14]	Privacy-preserving mutual authentication using ZKP and blockchain.	Formal privacy properties: identity hiding, unlinkability, traceability, and mutual authentication.	ZKP, permissioned blockchain, IoT authentication.	Reduce latency and test high-scale IoT traffic.
2025	*Zero-Trust Access Control Mechanism Based on Blockchain and Inner-Product Encryption in the Internet of Things in a 6G Environment* [22]	Blockchain-based Zero-Trust access control with inner-product encryption.	Formal access-control model with reputation, smart contracts, and encryption.	Blockchain, smart contracts, IPE, 6G-IoT.	Improve storage efficiency and fine-grained dynamic access control.
2025	*Blockchain-Based Secure Authentication Protocol for Fog-Enabled IoT Environments* [23]	Secure blockchain authentication protocol for fog-enabled IoT.	BAN logic, RoR model, AVISPA simulation, and DY/CK/eCK analysis.	Blockchain, fog-IoT, BAN, RoR, AVISPA.	Improve robustness against complex fog-IoT attack models.
2025	*Decentralized Identity Management for Internet of Things Devices Using IOTA Blockchain Technology* [16]	Decentralized IoT identity management using IOTA, DIDs, and VCs.	Architectural and experimental validation of DID/VC lifecycle in IoT.	IOTA, DIDs, VCs, IOTA Identity, Streams, Stronghold.	Optimize cryptography and test scalability in constrained devices.
2025	*ZT-IoTrust: A Quantum-Resistant Zero Trust Framework for Secure IoT Access Control* [24]	Quantum-resistant Zero-Trust IoT access control with federated trust evaluation.	LWE/Ring-LWE assumptions, Byzantine tolerance, and experimental validation.	Ring-LWE, lattice signatures, federated learning, edge IoT.	Improve side-channel resistance and resilience against stronger adversaries.
2025	*Blockchain-Integrated Secure Authentication Framework for Smart Grid IoT Using Energy-Aware Consensus Mechanisms* [25]	Blockchain authentication for smart-grid IoT with energy-aware consensus.	Simulation results: high accuracy, low delay, high TPS, and reduced validator energy.	Blockchain, DNN, smart-grid IoT, energy-aware consensus.	Add fault-tolerant consensus, federated learning, and hardware-in-the-loop tests.
2025	*Lightweight Blockchain for Authentication and Authorization in Resource-Constrained IoT Networks* [26]	Lightweight blockchain for IoT authentication and authorization.	Experimental validation of lower energy, latency, and attack-detection capability.	Lightweight blockchain, simplified PoS, constrained IoT.	Improve interoperability, scalability, and real-device deployment.
2025	*Intrusion Detection in IoT Networks Using Machine Learning and Deep Learning Approaches for MitM Attack Mitigation* [17]	MitM intrusion detection using LSTM, Random Forest, and SVM over IoT network-traffic data.	Empirical classifier evaluation; Random Forest reports 94% accuracy and LSTM 92% on the source dataset.	LSTM, Random Forest, SVM, prepared IoT network-traffic dataset.	Refine the models, explore RF–LSTM hybrid techniques, and address resource constraints for real-time IoT deployment.
2025	*Enhancing IoT Security: Assessing Instantaneous Communication Trust to Detect Man-in-the-Middle Attacks* [20]	IPCTCM for instantaneous communication-trust estimation using E2E delay to detect active MitM attacks.	Expected E2E delay is estimated with Kalman filtering and LSTM; the source testbed reports 98.9% attack-detection accuracy.	IoT laboratory testbed, Kalman filter, LSTM, E2E-delay measurements.	Broaden assessment of the trust model to other communication domains and deployment conditions.

**Table 2:** Global notation used throughout the manuscript.

Symbol	Meaning
$M_{i}$	IoT message submitted to the gateway.
$d_{i}$	Device identifier.
$\tau_{i}$	Session token.
$n_{i}$	Message/session nonce.
$t_{i}$, $T_{i}^{G}$	Credential/message timestamp and gateway receipt time, respectively.
$p_{i}$, $H(p_{i})$	Telemetry payload and its cryptographic digest.
$\sigma_{i}$	MAC or digital signature verified by the lower authentication layer.
${\mathrm{fw}}_{i}$, ${\mathrm{ctx}}_{i}$	Firmware-state evidence and trusted gateway-observed context.
$C_{i}$	Canonical post-authentication screening record.
$h_{i}$	Full cryptographic digest of the canonical screening record.
$\rho_{q}\left(h_{i}\right)=\left(a_{i},b_{i},c_{i}\right)$	Reduction in $h_{i}$ to the operational algebraic state in $Z_{q}^{3}$.
*q*, *s*	Algebraic modulus and translation parameter of the Yang–Baxter map.
$r_{s}$, $r_{12}$, $r_{23}$	Pairwise Yang–Baxter map and its actions on coordinates (1,2) and (2,3).
$L_{i}$, $R_{i}^{\mathrm{YB}}$	Final states of the left and right Yang–Baxter traversals.
$R_{\mathrm{chain}}$, $R_{\mathrm{fresh}}$, $R_{\mathrm{ctx}}$	Chain-proximity, freshness, and contextual risk components.
$R_{i}$	Aggregate deterministic operational-risk score.
α,β,γ	Non-negative risk weights satisfying α+β+γ=1.
$\theta_{\mathrm{risk}}$, $\theta_{\mathrm{revoke}}$	Challenge/risk and corroborated-revocation thresholds.
$\kappa_{i}=H\left(\mathrm{DID}\left(d_{i}\right)\right)$	Hashed DID key used by the local revocation cache and ledger registry.
$R_{\mathrm{cache}}$	Local gateway cache of published revocations.
$E_{i}$, $\ell_{i}$, $B_{j}$	Decision evidence, Merkle leaf, and anchored Merkle-batch root.
*B*, $T_{\mathrm{flush}}$, $t_{\mathrm{tx}}$	Merkle batch size, maximum flush interval, and modeled ledger commit time.

**Table 3:** Analytical batch-size sensitivity at 100 decisions/s and 2 s mean modeled commit time (fill-delay column ignores an earlier $T_{\mathrm{flush}}$ trigger).

*B*	$t_{\mathrm{tx}}/B$ (ms)	Logical Payload (B/Decision)	Anchors/1000	Mean Fill Wait (ms)
16	125.0	3.250	63	75
32	62.5	1.625	32	155
64	31.25	0.813	16	315
128	15.63	0.406	8	635
256	7.81	0.203	4	1275

**Table 4:** Formal claims, assumptions, and non-claims.

Property	Formal Support/Assumption	What Is Not Claimed
YBE correctness	Proposition 5 for all q≥2, $s\in Z_{q}$	YB equality is not identity/authenticity proof
Intermediate fault detection	Proposition 7, isolated nonzero single-path perturbation	No guarantee for common-mode or comparator compromise
Authentication preservation	Propositions 12 and 13	No security if the underlying authenticator is itself broken
Risk normalization	Proposition 2	No learned probability-of-compromise interpretation
Adaptive threshold safety	Proposition 8	No complete long-horizon poisoning proof
Audit tamper evidence	Proposition 9, collision resistance and authenticated root	No completeness for evidence suppressed before submission
Revocation convergence	Propositions 10 and 11	No deterministic bound during partitions or missed events
Incremental complexity	Proposition 15	Operation count is not battery-energy measurement

**Table 5:** Comparative evaluation framework.

Approach	Security Kernel	Main Detection Principle	Evaluation Role
Ali and Al-Sharafi [17]	Reported RF-based ML profile	Traffic-feature classification for MitM detection	Learning-based baseline
Basri et al. [20]	Reported IPCTCM delay-trust profile	Communication-delay trust estimation	Instantaneous trust baseline
Proposed YB-IoT-CG	Deterministic Yang–Baxter credential kernel with blockchain anchoring	Algebraic credential consistency, deterministic acceptance/rejection, on-chain revocation	Proposed lightweight kernel

**Table 6:** Experimental factors, levels, and simulation parameters.

Factor	Level/Value	Parameter	Purpose
Number of runs	60 repetitions	RUNS = 60	Generate stable statistical distributions
Packets per run	1000 packets	PACKETS_PER_RUN = 1000	Model repeated IoT telemetry transmission
Payload size	256 bytes	PAYLOAD_BYTES = 256	Estimate useful delivered telemetry
Attack rate	30% malicious packets	ATTACK_RATE = 0.30	Represent MitM pressure over the packet stream
Observation window	10 s	WINDOW_SECONDS = 10	Normalize goodput in bytes per second
Compared kernels	Ali-RF, Basri-IPCTCM, YB-IoT-CG	SYSTEMS	Evaluate learning, delay-trust, and deterministic algebraic approaches
Normal delay	Mean 80 ms, std. 10 ms	N(0.080,0.010)	Model legitimate IoT communication delay
MitM extra delay	Mean 180 ms, std. 35 ms	N(0.180,0.035)	Model additional delay caused by interception or manipulation
YB modulus	997	q=997	Finite *non-cryptographic* algebraic domain; the full 256-bit digest remains the security/audit binding
Anchoring batch size	64 evidence records	BATCH_B = 64	Amortize ledger transactions over decisions
Ledger commit latency	Mean 2.0 s, std. 0.4 s	N(2.0,0.4)	Model permissioned-ledger finality [6]
Event propagation delay	Mean 150 ms, std. 40 ms	N(0.150,0.040)	Model revocation-event fan-out to gateways

**Table 7:** Risk-policy parameters used in the reported simulation.

Parameter	Exact Simulation Value	Role/Update Rule
α	0.15	Weight of $R_{\mathrm{chain}}$
β	0.60	Weight of $R_{\mathrm{fresh}}$
γ	0.25	Weight of $R_{\mathrm{ctx}}$; α+β+γ=1
$\theta_{\mathrm{risk}}\left(0\right)$	0.60	Initial challenge/risk threshold
$\theta_{\mathrm{revoke}}$	0.85	Corroborated-revocation threshold; fixed during the deployment epoch
$\theta_{\min},\theta_{\max}$	0.50, 0.70	Bounds for adaptive $\theta_{\mathrm{risk}}$
$\delta_{\mathrm{clk}}$	20 ms	Tolerated clock-skew bound
$\Delta_{\mathrm{soft}}$	100 ms	Soft timestamp-age limit
$\Delta_{\mathrm{hard}}$	200 ms	Hard timestamp-age limit
*W*	10 s	Active authenticated-nonce replay window
$\xi_{\ell }$	0.50forallℓ	Context-severity threshold(s) used for corroboration
$w_{\ell }$	1/mforallℓ	Context-predicate weights, with $\sum_{\ell }w_{\ell }=1$
λ	0.05	EWMA coefficient
η	0.01	Maximum absolute threshold change per accepted update
*J*	256	Number of deterministic endpoint-index buckets
*K*	8	Maximum risky endpoints inspected in the queried bucket
Initial replay state	Empty authenticated-nonce history	Initial state of $N_{d}\left(W\right)$ at the start of each run
Initial endpoint/registry state	Fixed preloaded endpoint set; empty revocation cache	Initial risky-endpoint and revocation-cache state
Threshold update frequency	After each authenticated Accept	$\theta_{\mathrm{risk}}$ is updated only after an authenticated accepted message; challenged, revoked, and unauthenticated messages do not update it

**Table 8:** Descriptive packet-level simulator outputs over 60 seeded runs: median [IQR] and bootstrap 95% CI of the median.

Metric	Ali-RF Simulator Profile	Basri-IPCTCM Simulator Profile	YB-IoT-CG
Goodput (B/s)	17,356.8 [17,120.0–17,664.0] CI (17,267.2–17,561.6)	17,497.6 [17,126.4–17,670.4] CI (17,305.6–17,561.6)	17,894.4 [17,555.2–18,060.8] CI (17,650.9–17,945.6)
Decision delay (ms)	4.396 [4.379–4.420] CI (4.388–4.406)	89.957 [89.495–90.695] CI (89.793–90.357)	0.0078 [0.0078–0.0079] CI (0.0078–0.0078)
Kernel complexity (ops/packet)	1251.0 [1246.7–1254.6] CI (1248.4–1253.0)	420.2 [419.0–421.6] CI (419.7–421.0)	40 [40–40] CI (40–40)
Aggregate decision accuracy (%)	96.50 [96.10–96.90] CI (96.20–96.70)	98.10 [97.78–98.50] CI (97.95–98.20)	99.40 [99.30–99.50] CI (99.40–99.40)
False rejection rate (%)	2.83 [2.45–3.44] CI (2.75–3.02)	2.70 [2.17–3.19] CI (2.51–2.93)	0.43 [0.29–0.58] CI (0.42–0.56)

**Table 9:** Modeled ledger-layer overhead over 60 seeded runs (median [IQR]).

Metric	Value	Interpretation
Amortized ledger service-time equivalent	31.4 [26.7–35.5] ms	$t_{\mathrm{tx}}/B$ for B=64; not individual verification latency
Logical anchor payload per decision	0.81 [0.81–0.81] B	root + modeled metadata only; physical ledger overhead excluded
Anchor transactions per 1000 packets	16 [16–16]	⌈1000/64⌉ after final flush
Modeled revocation propagation time	2.15 [1.88–2.42] s	empirical $T_{c}+T_{e}$ distribution; not a hard deterministic bound
Revocation transactions per run	292 [286–305]	confirmed simulated adversarial credentials under the declared policy
Audit completeness after final flush	100%	all decision branches generate evidence in the simulator
Synchronous ledger operations on packet path	0	asynchronous audit; local cache lookup only

## Data Availability

The complete simulation artifact—a single Python notebook that deterministically regenerates every figure, table, and statistical test in this article from the master seed 20260705, together with the seed manifest, per-run raw results, and recorded software environment—is available from the corresponding author upon reasonable request and is intended for public release upon acceptance.

## Abbreviations

The following abbreviations are used in this manuscript:

ABAC	Attribute-Based Access Control
AI	Artificial Intelligence
CoAP	Constrained Application Protocol
DID	Decentralized Identifier
DTLS	Datagram Transport Layer Security
EVM	Ethereum Virtual Machine
IoT	Internet of Things
IPCTCM	Instantaneous Packet Communication Trust Calculation Model
MAC	Message Authentication Code
MitM	Man-in-the-Middle
MQTT	Message Queuing Telemetry Transport
PKI	Public Key Infrastructure
PUF	Physically Unclonable Function
TEE	Trusted Execution Environment
TLS	Transport Layer Security
TMTO	Time–Memory Trade-Off
VC	Verifiable Credential
YB	Yang–Baxter
YBE	Yang–Baxter Equation
YB-IoT-CG	Yang–Baxter IoT Consistency Gateway
ZKP	Zero-Knowledge Proof
